# EP300 and SIRT1/6 Co-Regulate Lapatinib Sensitivity Via Modulating FOXO3-Acetylation and Activity in Breast Cancer

**DOI:** 10.3390/cancers11081067

**Published:** 2019-07-28

**Authors:** Zimam Mahmud, Ana R. Gomes, Hee Jin Lee, Sathid Aimjongjun, Yannasittha Jiramongkol, Shang Yao, Stefania Zona, Glowi Alasiri, Gyungyub Gong, Ernesto Yagüe, Eric W.-F. Lam

**Affiliations:** 1 Department of Surgery and Cancer, Imperial College London, Imperial Centre for Translational and Experimental Medicine (ICTEM), Du Cane Road, London W12 0NN, UK; 2 Department of Pathology, Asan Medical Center, University of Ulsan College of Medicine, Seoul 05505, Korea; 3 Multidisciplinary Unit, Faculty of Science, Mahidol University, Phayathai, Rajdhevi, Bangkok 10400, Thailand

**Keywords:** acetylation, breast cancer, drug resistance, EP300, sirtuins, FOXO3, lapatinib, post-translational modifications

## Abstract

Forkhead Box O3 (FOXO3) is a tumor suppressor whose activity is fine-tuned by post-translational modifications (PTMs). In this study, using the BT474 breast cancer cells and a recently established lapatinib resistant (BT474-Lap^R^) cell line, we observed that higher FOXO3 and acetylated (Ac)-FOXO3 levels correlate with lapatinib sensitivity. Subsequent ectopic expression of EP300 led to an increase in acetylated-FOXO3 in sensitive but not in resistant cells. Drug sensitivity assays revealed that sensitive BT474 cells show increased lapatinib cytotoxicity upon over-expression of wild-type but not acetylation-deficient EP300. Moreover, FOXO3 recruitment to target gene promoters is associated with target gene expression and drug response in sensitive cells and the inability of FOXO3 to bind its target genes correlates with lapatinib-resistance in BT474-Lap^R^ cells. In addition, using SIRT1/6 specific siRNAs and chemical inhibitor, we also found that sirtuin 1 and -6 (SIRT1 and -6) play a part in fine-tuning FOXO3 acetylation and lapatinib sensitivity. Consistent with this, immunohistochemistry results from different breast cancer subtypes showed that high SIRT6/1 levels are associated with constitutive high FOXO3 expression which is related to FOXO3 deregulation/inactivation and poor prognosis in breast cancer patient samples. Collectively, our results suggest the involvement of FOXO3 acetylation in regulating lapatinib sensitivity of HER2-positive breast cancers.

## 1. Introduction

Breast cancer is the most common malignancy in women and the principal cause of cancer death worldwide [1]. FOXO proteins are Forkhead Box (class O) transcription factors that are evolutionarily conserved from invertebrates to mammals, with invertebrates having only one *FOXO* gene and mammals four- *FOXO1, FOXO3, FOXO4* and *FOXO6* [2]. FOXO3 is a tumor suppressor often deregulated in cancers, including glioblastoma, leukemia and breast and prostate cancer, due to its role in restricting cell proliferation and promoting cell death [3]. Once activated, FOXO3 proteins bind to the conserved sequence motif GTAAA(C/T)A to induce transcription of their target genes [4,5]. The biological activity of FOXO3 is predominantly regulated by post-translational modifications (PTMs) in response to different environmental and intracellular stimuli [6]. These PTMs include phosphorylation, acetylation, methylation, ubiquitination and glycosylation, which subsequently control FOXO3 protein expression, subcellular localization, DNA binding ability, transcriptional activity and stability [6,7].

Among all FOXO PTMs, phosphorylation is the best studied. Upon activation of phosphatidylinositol 3-kinase-protein B kinase (PI3K-PKB/AKT) signaling pathway, phosphorylation of human FOXO3 at three conserved sites (T32, S253 and S315) by AKT creates binding sites for the scaffolding protein 14-3-3, which in turn sequesters the phosphorylated protein in the cytoplasm and ultimately leads to their proteasomal degradation [3,8,9].

Another important PTM of FOXO3 but as yet not well-studied, is acetylation. Protein acetylation is regulated by the opposing actions of the acetyl-transferases [e.g., EP300 or (cAMP response element binding (CREB) binding protein (CBP)] and the de-acetylases, such as sirtuins (SIRTs). Oxidative stress and the formation of reactive oxygen species (ROS) induce FOXO3 acetylation on lysine residues K242, K245 and K259 [10], which are found within the DNA binding domain of the FOXO protein. In turn, acetylation can affect the DNA binding ability and transcriptional activity of FOXO3. However, it is still under debate as to whether this modification causes an increase or a decrease in the affinity of FOXO3 for DNA [2,11]. Nonetheless, acetylation has also been suggested to switch target gene expression from cell cycle arrest to apoptosis and increase the nuclear concentration of FOXO proteins [7,12]. Conversely, de-acetylation of FOXO3 has been suggested to promote expression of genes implicated in cell cycle arrest and stress resistance [8].

Lapatinib is a small molecule inhibitor administered to patients with metastatic breast cancer over-expressing the human epidermal growth factor receptor 1/2 (EGFR1/HER2) [13]. It is a dual tyrosine kinase inhibitor (TKI) that inhibits downstream signaling initiated by the epidermal growth factor (EGF) receptor (EGFR, also known as *HER1*) and HER2 [13,14]. However, resistance to lapatinib often arises through mechanisms, including increased drug efflux, enhanced pro-survival pathways and over-activation of PI3K-AKT signaling pathway [4]. In this study, we used the *HER2* over-expressing breast cancer cell line, BT474 and a lapatinib resistant derivative (BT474 Lap^R^), in an effort to investigate the functional outcome of FOXO3 acetylation as well as the role of FOXO3 acetylation in modulating lapatinib response.

## 2. Results

### 2.1. Lapatinib Treatment Increases FOXO3 Acetylation and Its Activity in Sensitive BT474 Cells

**Figure 1:**
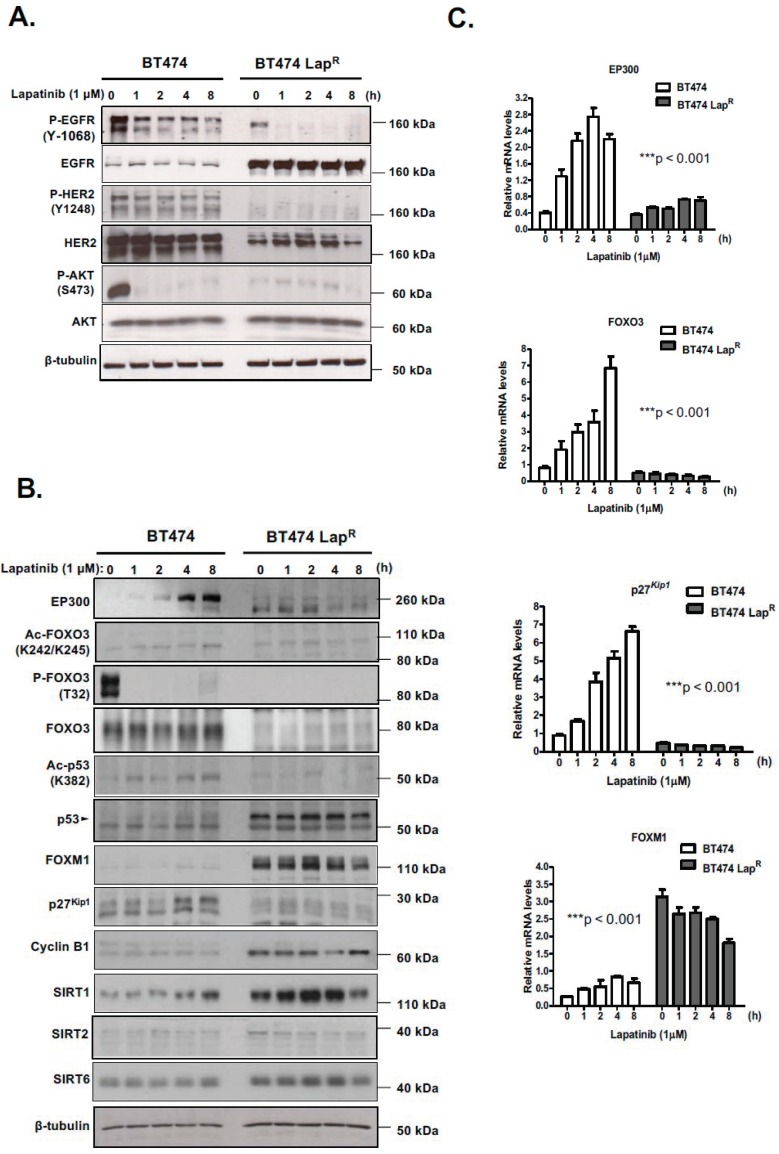
Lapatinib treatment increases acetylation of FOXO3 as well as its expression and transcriptional activity in BT474 sensitive cells but not in resistant cells. (**A**,**B**) The sensitive (BT474) and lapatinib resistant BT474 Lap^R^ cells were either left untreated or treated with 1 µM lapatinib for the mentioned time points. Protein lysates from whole-cell extracts were then analyzed by western blotting using the antibodies against the proteins indicated. Molecular weight markers are shown. (**C**) The mRNA levels of *EP300*, *FOXO3*, *p27^kip1^* and *FOXM1* were assessed by real time qPCR. The RPL19 housekeeping gene was used as a control to normalize gene expression. The kinetics of *EP300*, *FOXO3*, *p27^kip1^* and *FOXM1* mRNA expression were analyzed by two-way ANOVA and found to be significantly different between BT474 and BT474 Lap^R^ cells. Data are representative of at least 3 independent experiments. Data represent means ± SEM (*** *p*  <  0.001, for all mRNA species, respectively).

In an effort to better characterize the role of EP300-mediated regulation of FOXO3 in mediating lapatinib response, we evaluated the effects of the dual tyrosine kinase inhibitor lapatinib on FOXO3 acetylation and activity in BT474 cells, which overexpress HER2, as well as BT474-Lap^R^ cells, a recently developed BT474 lapatinib-resistant derivative (Supplementary Figure S1). Lapatinib activity was confirmed in both cells, since 1 h treatment with 1 µM lapatinib notably reduced EGFR phosphorylation without changing its expression (Figure 1A) (Supplementary Figure S8A). Similar effects were observed with HER2 in BT474 cells, although HER2 expression was diminished in BT474-Lap^R^ cells, probably during the acquisition of resistance. Lapatinib also inhibited AKT phosphorylation in BT474 cells, as AKT acts downstream from HER2, whereas it did not have any effect on resistant cells.

Lapatinib induced expression of EP300 and FOXO3, an effect observed at both the mRNA and protein levels and efficiently inhibited FOXO3 phosphorylation (T32), due to its inhibition of AKT, in BT474 cells but not in BT474-Lap^R^ cells (Figure 1B,C) (Supplementary Figure S8B). Importantly, both FOXO3 and p53 acetylation in BT474 cells followed similar kinetics as EP300 expression, indicating that they are good markers for EP300 acetyl-transferase activity [15]. As expected, there were no acetylation changes in BT474-Lap^R^ cells. Notably, high levels of acetylated FOXO3 after lapatinib treatment in BT474 cells positively correlated with the expression of its downstream transcriptional target p27^Kip1^. at both mRNA and protein levels (Figure 1B,C). Moreover, high levels of FOXM1 expression were found in resistant cells at both mRNA and protein levels (Figure 1B,C). FOXM1 is an oncogene repressed by FOXO3 activity and has a role in inducing lapatinib resistance [16,17]. Lower levels of EP300 and higher levels of the nuclear deacetylases, sirtuins (e.g., SIRT1 and SIRT6) were also detected in the BT474-Lap^R^ cells, which might also contribute to the reduced FOXO3 activity in BT474-Lap^R^ cells and therefore lapatinib resistance in HER2-positive breast cancer cells (Figure 1B). Notably, another sirtuin SIRT2, which can shuttle between the cytoplasm and the nucleus, was found to be expressed but at low and similar levels in the lapatinib sensitive and resistant BT474 cells, suggesting that it might not have a predominant role in mediating FOXO3 acetylation and lapatinib resistance. Thus, lapatinib induces EP300 expression and acetylation and FOXO3 acetylation in BT474 cells.

### 2.2. Lapatinib Induces FOXO3 Recruitment to Its Target Gene Promoters in Sensitive but not in Lapatinib-Resistant Cells

**Figure 2:**
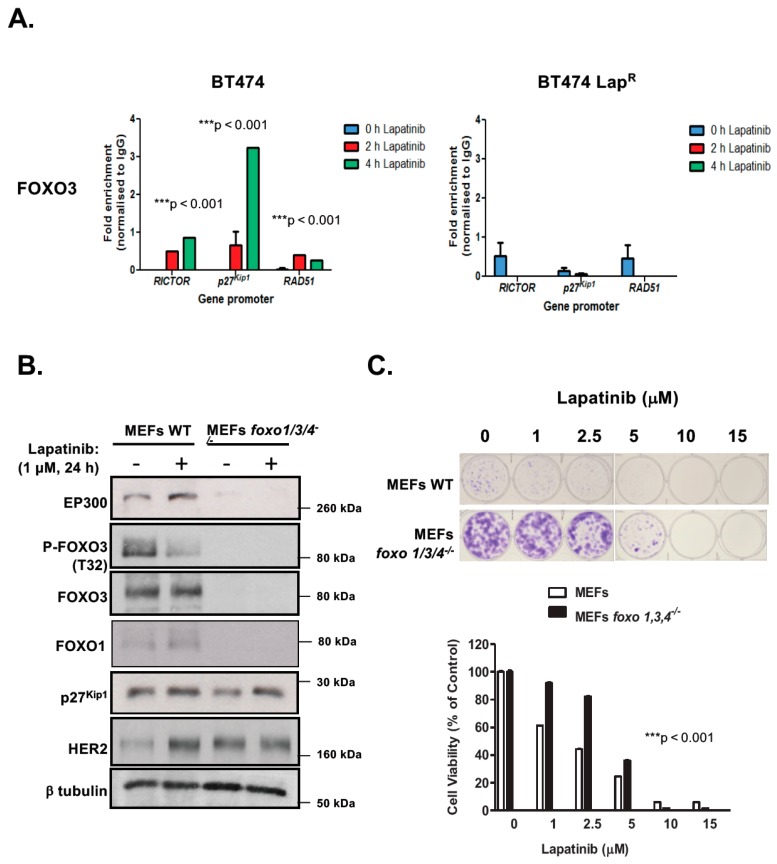
FOXO3 regulates lapatinib sensitivity in BT4T4 and MEFs. (**A**) Lapatinib induces Figure 3. recruitment to its target gene promoters in BT474 cells. Sensitive (WT) and drug resistant BT474 Lap^R^ cells were harvested following treatment with 1 µM lapatinib for 0, 2 or 4 h. FOXO3 binding to the gene promoters of *RICTOR*, *p27^Kip1^* and *RAD51* was then assessed by Chromatin immunoprecipitation (ChIP) analysis using anti-FOXO3 antibody. After crosslink reversal, immunoprecipitated DNA was amplified by qPCR using primers for each gene promoter indicated. Data were analyzed using the fold enrichment method and bars represent the means ± SEM of three independent experiments (*n* = 3). The kinetics of FOXO3-binding were analyzed by two-way ANOVA and found to be significantly different between BT474 and BT474 Lap^R^ cells (*** *p*  <  0.001, for all genes, respectively). (**B**,**C**) FOXO3 regulates lapatinib sensitivity in MEFs. (**B**) Western blot analysis was performed to analyze the protein levels of EP300, FOXO3, FOXM1 and others indicated in MEFs and *Foxo1,3,4*^−/−^ MEFs. Representative western blots are shown. (**C**) Wild-type MEFs and *Foxo1,3,4*^−/−^ MEFs were treated with increasing doses of lapatinib and their sensitivity to lapatinib was assessed by clonogenic assay. Their clonogenicity in response to lapatinib was analyzed by two-way ANOVA and found to be significantly different (*** *p*   < 0.001) from one another.

We have previously demonstrated that FOXO3 activates expression of target genes which orchestrate a transcriptional program leading to lapatinib sensitivity [16,17,18,19,20]. We next sought to determine whether FOXO3 is recruited to the promoters of its target genes in response to lapatinib treatment. To this end, we used chromatin immunoprecipitation (ChIP) to determine the recruitment of endogenous FOXO3 to its transcriptional targets *RICTOR*, *p27^Kip1^* and *RAD51* [9,21,22]. FOXO3 displayed low binding to its target genes in untreated BT474 cells (Figure 2A), which increased upon lapatinib exposure. FOXO3 had the maximum binding at 4 h post-lapatinib treatment, with the exception of *RAD51* which was induced with faster kinetics 2 h after drug exposure. Conversely, FOXO3 binding was very weak in untreated BT474-Lap^R^ cells and after lapatinib treatment. Collectively, these results suggest that lapatinib responses are mediated by FOXO3 binding and subsequent activation of its target genes in BT474 but not in lapatinib-resistant cells.

### 2.3. Triple foxo1/3/4 Knock-out Mouse Embryonic Fibroblasts Are Resistant to Lapatinib

Previous studies have shown that FOXO proteins are functionally redundant and can compensate for the loss of one another [23]. To ascertain the role of FOXO3 on lapatinib response, we studied the effects of lapatinib treatment on wild-type (WT) and *foxo1/3/4*^−/−^ MEFs. Western blot analysis showed high levels of HER2 expression in *foxo1/3/4*^−/−^ MEFs (Figure 2B) (Supplementary Figure S8C), suggesting that they might be susceptible to the antiproliferative function of lapatinib. Interestingly, both EP300 and FOXO3 were practically absent upon *foxo1/3/4* knock-out and, as expected, the direct FOXO3 target p27^Kip1^ was down-regulated. As previously found in BT474 cells, when MEFs were treated with 1 µM lapatinib for 24 h, EP300 expression was up-regulated and FOXO3 phosphorylation (T32) decreased, with a corresponding increase in p27^Kip1^ levels. Contrary to BT474 cells, which have high levels of endogenous HER2, naive MEFs showed very low levels of HER2. Upon lapatinib treatment, HER2 levels increased notably. Next, we assess how long-term viability of triple *foxo1/3/4* knock-out MEFs was affected by lapatinib using clonogenic assays. Knock-out cells formed more robust colonies in the absence of the drug, probably as an indication of their increased transformation, that were less susceptible to lapatinib treatment in the 1–5 µM range (Figure 2C). Thus, *triple Foxo1/3/4* knock-out MEFs are more resistant to lapatinib. To further demonstrate that FOXO3 plays a key role in lapatinib response, we studied the effects of reintroduction of FOXO3 in the BT474-Lap^R^ and the triple *foxo1/3/4* knock-out MEFs (Supplementary Figure S2). Overexpression of wild-type (unphosphorylated) FOXO3 in these FOXO3-depleted cells had similar effects as treatment of the BT474 and WT MEFs with lapatinib, as FOXO3 is the target of lapatinib in the wild-type BT474 and MEFs. Specifically, overexpression of FOXO3 caused a loss of clonogenicity in both the BT474-Lap^R^ and the triple *foxo1/3/4* knock-out MEFs. Furthermore, increased lapatinib dosages caused additional decreases in clonogenicity, further supporting the idea that FOXO3 is a key target of lapatinib and downregulation of FOXO3 expression causes lapatinib resistance.

### 2.4. EP300-FOXO3 Interaction Is Induced by Lapatinib Treatment in BT474 Cells

**Figure 3:**
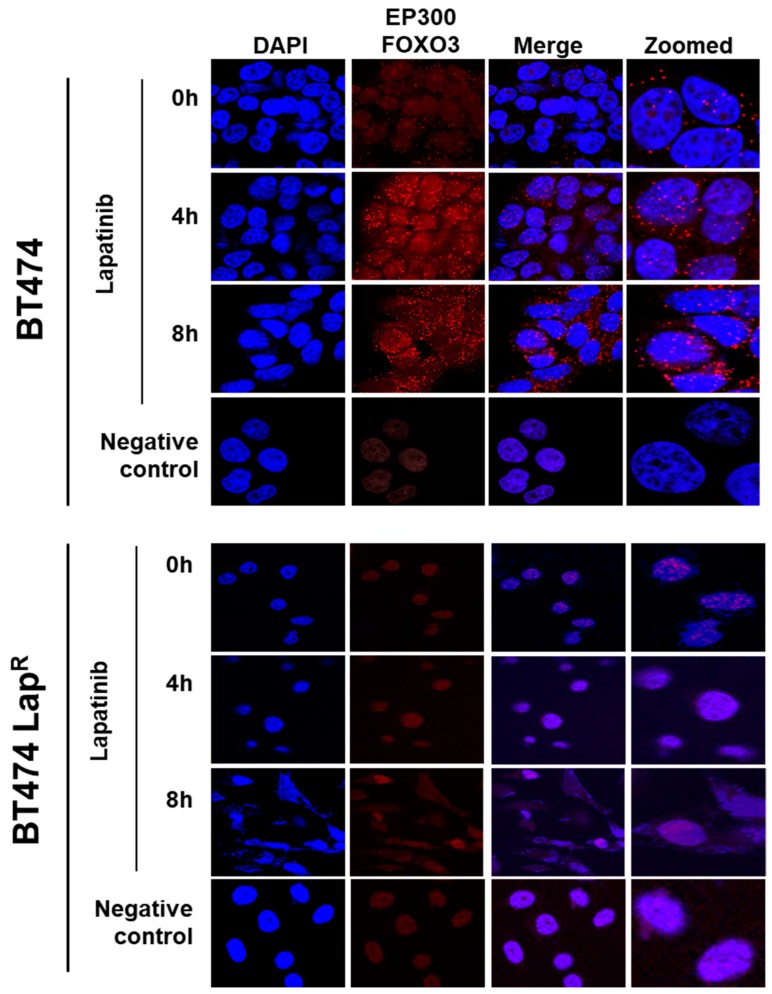
Duolink proximity ligation assay (PLA) for detecting the protein-protein interactions between EP300 and FOXO3 in BT474 and BT474 Lap^R^ cells. BT474 and BT474 Lap^R^ cells were treated with 1 µM lapatinib for 0, 4 and 8 h. Cells without EP300 and FOXO3 antibodies were used as negative control. Cells were visualized with a Leica TCS SP5 confocal microscope equipped with a 63X oil immersion objective and LAS-AF software. Each red spot represents a single interaction and DNA was stained with 4, 6-diamidino-2-phenyl indole (DAPI) in blue. Scale bar: 10 µm. Representative confocal images are shown from three independent experiments.

To investigate the possible interaction between FOXO3 and EP300, a proximity ligation assay (PLA) was performed. Lapatinib treatment induced EP300-FOXO3 interaction in a time-dependent manner in BT474 cells as more red specks were observed, both in the nucleus and the perinuclear region, after 8 h treatment (Figure 3). On the other hand, no interaction was observed in BT474-LapR cells (Figure 3). This suggests that lapatinib induces interaction between FOXO3 and EP300 in sensitive but not in resistant BT474 cells.

### 2.5. EP300 Acetylates FOXO3 and Induces Lapatinib Sensitivity

**Figure 4:**
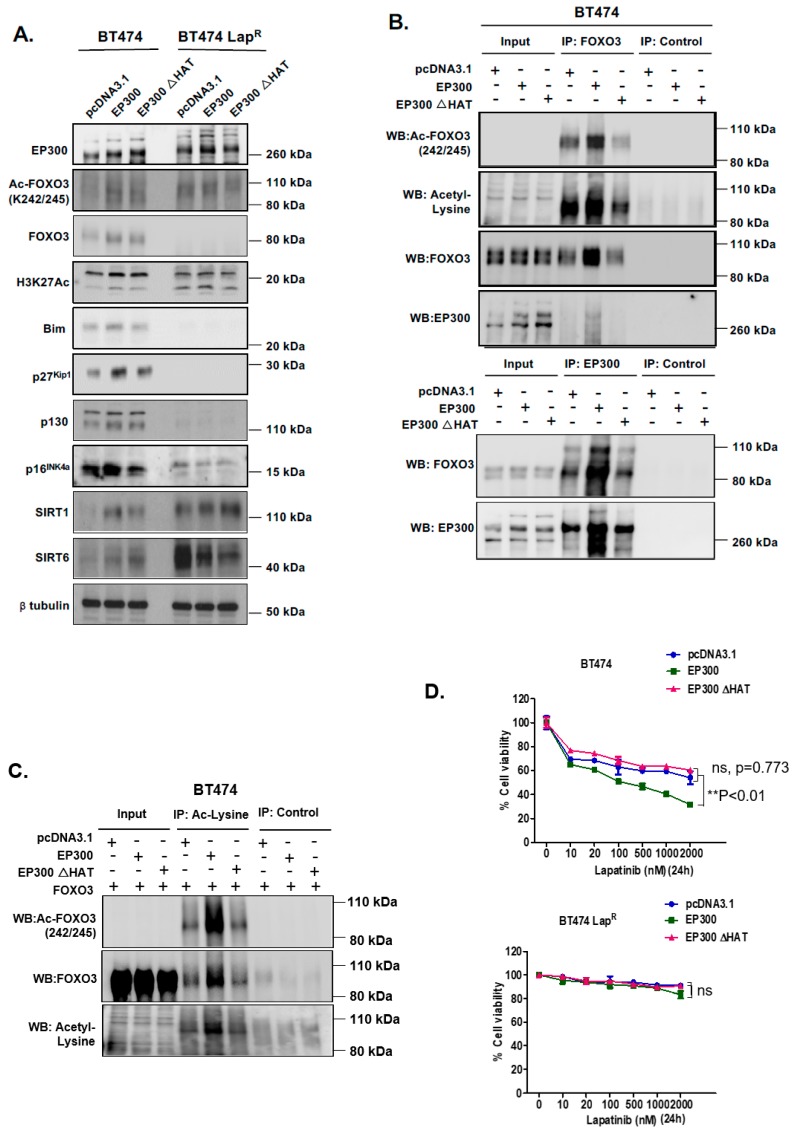
EP300 overexpression upregulates FOXO3 acetylation/activity and increases lapatinib sensitivity. (**A**) BT474 and BT474-Lap^R^ cells were transiently transfected with the empty vector pcDNA3.1, the plasmid encoding wild-type EP300 or the one encoding for its acetyl-transferase mutant derivative (EP300 ΔHAT). Proteins were obtained from whole cell extracts after 24 h following transfection. Western blotting was performed using the protein lysates to assess the expression levels of EP300, FOXO3 and other indicated proteins. (**B**) Protein lysates of BT474 cells transiently transfected with EP300 or EP300 ΔHAT obtained were assessed by Co-immunoprecipitation (Co-IP) with an anti-FOXO3 antibody. Subsequent immunoblotting was performed using antibodies against Ac-Lys, Ac-FOXO3 and FOXO3. The anti-IgG was used as a negative control. (**C**) Reverse-Co-IP was performed with anti-Acetyl Lysine antibody using the protein lysates of BT474 cells transiently transfected with EP300 and FOXO3 or EP300 ΔHAT and FOXO3. Subsequent immunoblotting was performed using antibodies against Ac-Lys, Ac-FOXO3 and FOXO3. The anti-IgG was used as a negative control. (**D**) Transiently transfected BT474 WT cells were seeded in 96-well plates and treated with lapatinib at a range of concentrations from 10 to 2000 nM. Twenty-four hours after treatment, cells were fixed and stained with the protein-binding dye SRB. Values obtained were normalized against the corresponding untreated controls and presented as percentages. Data represent means ± SEM (2-way ANOVA; non-significant, ns; significant, ** *p* < 0.01).

To further investigate the role of EP300-mediated acetylation of FOXO3 upon lapatinib treatment, we used an acetyl-transferase-defective mutant (HAT mutation at H1415A E1423A Y1424A L1428S Y1430A H1434A) to transiently transfect BT474 and BT474-Lap^R^ cells. Western blot analysis indicated that upon transfection of wild-type EP300 in BT474 cells there was an upregulation of FOXO3 expression, while the upregulation of FOXO3 was comparatively weaker upon transfection of the EP300 HAT mutant (Figure 4A) (Supplementary Figure S8D). However, this EP300-dependent FOXO3 up-regulation did not occur in BT474-Lap^R^ cells. As expected, acetylation of FOXO3 (K242/245) and histone 3 (K27) were induced upon wild-type EP300 transfection in BT474 cells but this induction was comparatively weaker upon transfection of the HAT mutant. No changes in FOXO3 or histone 3 acetylation were observed in BT474-Lap^R^ cells. These data were further confirmed by co-immunoprecipitation experiments in BT474 cells. The FOXO3-EP300 interaction that we had previously detected by PLA (Figure 3) was reconfirmed when pulling-down using either FOXO3 (Figure 4B, upper panel) or EP300 (Figure 4B, lower panel) antibodies, as the intensity of the EP300 and FOXO3 bands, respectively, upon transfection of wild-type EP300 were stronger than with the HAT mutant. As expected, FOXO3 acetylation (K242/245) increased upon transfection with the wild-type but not with the EP300 HAT mutant (FOXO3 pull-down and acetylated FOXO3 detection; Figure 4B, upper panel). Similarly, when reverse co-immunoprecipitation was used to pull-down proteins with acetylated lysines, acetylated FOXO3 (K242/245) was clearly detectable upon transfection with FOXO3 and wild-type EP300 but not with the EP300 HAT mutant (Figure 4C). Consistently, ectopic expression of wild-type EP300 induced the expression of downstream FOXO3 target genes Bim, p27^Kip1^, p130 and p16^INK4a^ in BT474 but not in BT474-Lap^R^ cells (Figure 4A). Importantly, up-regulation of FOXO3 target genes did not occur upon transfection of the EP300 HAT mutant. This further confirms the positive regulatory role of EP300-mediated acetylation on FOXO3 transcriptional activity.

Since EP300 overexpression can induce FOXO3 activity and its downstream antiproliferative targets in parental but not the BT474-Lap^R^ resistant cells, we next performed sulphorhodamine B (SRB) assays to assess lapatinib sensitivity upon EP300 transfection. Over-expression of wild-type EP300 but not the acetyl-transferase deficient HAΤ EP300 mutant, increased lapatinib sensitivity in BT474 cells (Figure 4D, upper panel). Notably, ectopic expression of EP300 HAΤ mutant led to a slight but not significant increase in cell viability in sensitive BT474 cells upon lapatinib treatment, suggesting that the acetyl-transferase deficient EP300 mutant can titer the normal acetyl-transferase activity, as previously described for truncated mutants of EP300 [24]. On the contrary, EP300 over-expression in BT474-Lap^R^ cells did not result in any significant effect on lapatinib response until lapatinib reached 2 μM (Figure 4D, lower panel; Supplementary Figure S3) and this could be due to the high levels of deacetylases (e.g., SIRTs). Similar results are also observed for the lapatinib sensitive and resistant SKBR3 and SKBR3-Lap^R^ breast cancer cell lines (Supplementary Figure S4). These data confirms that EP300 acetylates FOXO3 and leads to an increase in lapatinib sensitivity.

### 2.6. EP300 Regulates Long-Term Lapatinib Sensitivity in Wild-Type BT474 but not in BT474-Lap^R^ Cells

**Figure 5:**
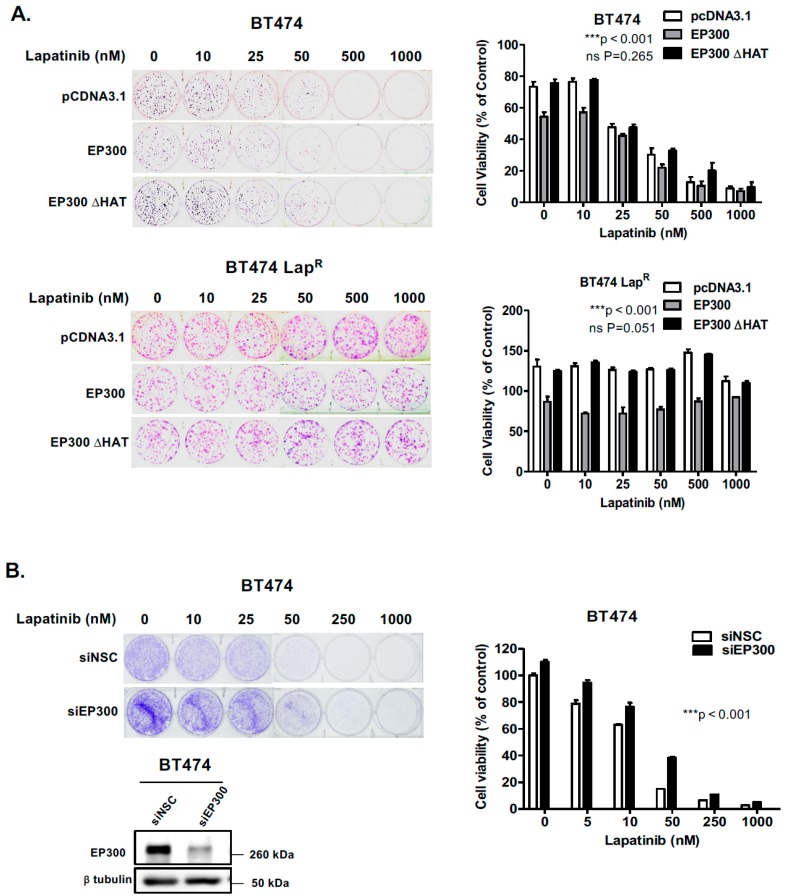
EP300 expression correlates with increased long-term lapatinib cytotoxicity in sensitive but not BT474 Lap^R^ cells. (**A**) BT474 and lapatinib resistant BT474 Lap^R^ cells were seeded 2000 and 1000 cells per well, respectively, in 6-well plates and transfected with the empty vector pcDNA3.1, wild-type EP300 or EP300 ΔHAT mutant expressing vector. (**B**) BT474 cells were seeded 2000 cells per well and transfected with non-targeting control (siNSC) or EP300-targeting siRNA pools. The transfected cells were then allowed to grow overnight and then treated with the lapatinib concentrations indicated (0,10, 25, 50, 500 and 1000 nM). Western blot analysis demonstrated EP300 depletion (lower panel). Twenty-four hours following treatment the medium was changed and colony formation was allowed for 28 and 14 days in sensitive and resistant BT474 cells, respectively. Cells were then fixed with 4% para formaldehyde and stained with crystal violet. The stain was solubilized with 33% acetic acid and absorbance obtained at 592 nm. Values obtained were converted to percentage of viability with untreated cells carrying the empty vector set to 100% viability. Data were finally normalized to the respective untreated empty-vector transfected cells. Bars represent the mean ± SEM of three independent transfection experiments (*n* = 3, R = 3) and statistical analysis was performed using ANOVA analysis (* *p* < 0.05, ** *p* < 0.01, *** *p* < 0.001) comparing the EP300, EP300 ΔHAT or siEP300-transfected cells versus controls.

Changes is short-term drug sensitivity may lead to long-term proliferation properties important in cancer treatment. In order to ascertain whether the short-term data shown above had any effect on the long-term lapatinib viability of BT474 cells, we performed clonogenic assays 24 h after transfection. Indeed, EP300 overexpressing cells showed increased lapatinib sensitivity when compared with control cells transfected with the empty vector or the acetyl-transferase defective mutant (Figure 5A,B) (Supplementary Figure S8E). These results suggest that EP300-mediated acetylation has a role in lapatinib-mediated cytotoxicity in sensitive BT474 cells. Noteworthy, the resistant cells over-expressing EP300 had lower clonogenicity compared to the control cells, suggesting that EP300 is involved in long-term cell viability and self-renewal but does not affect drug sensitivity in BT474-Lap^R^ cell lines (Figure 5A) (Supplementary Figure S8E). To examine further the role of EP300 in lapatinib sensitivity, siRNA-mediated knockdown of EP300 was performed in BT474 cells. The results showed a significant elevation of long-term cell viability around 50 nM of lapatinib after silencing EP300 (Figure 5B). Thus, acetylation of FOXO3 by EP300 leads to long-term sensitivity to lapatinib. The fact that overexpression of EP300 has no effect on FOXO3 acetylation and lapatinib-sensitivity in the BT474-Lap^R^ further suggests that FOXO3 acetylation is important for lapatinib sensitivity and also indicates that FOXO3 acetylation is also modulated by other regulators, including the deacetylases, SIRTs (Sirtuins). Notably, overexpression of EP300 decreased the clonogenicity of the BT474-Lap^R^ cells, suggesting other EP300 targets are also involved in the renewal of the BT474-Lap^R^ cells but they are unlikely to have a predominant role in lapatinib-sensitivity as EP300 expression has no effects on lapatinib sensitivity in the resistant cells.

### 2.7. Silencing of SIRT1 and SIRT6 Correlates with Increased FOXO3 Acetylation and Its Transcriptional Activity

**Figure 6:**
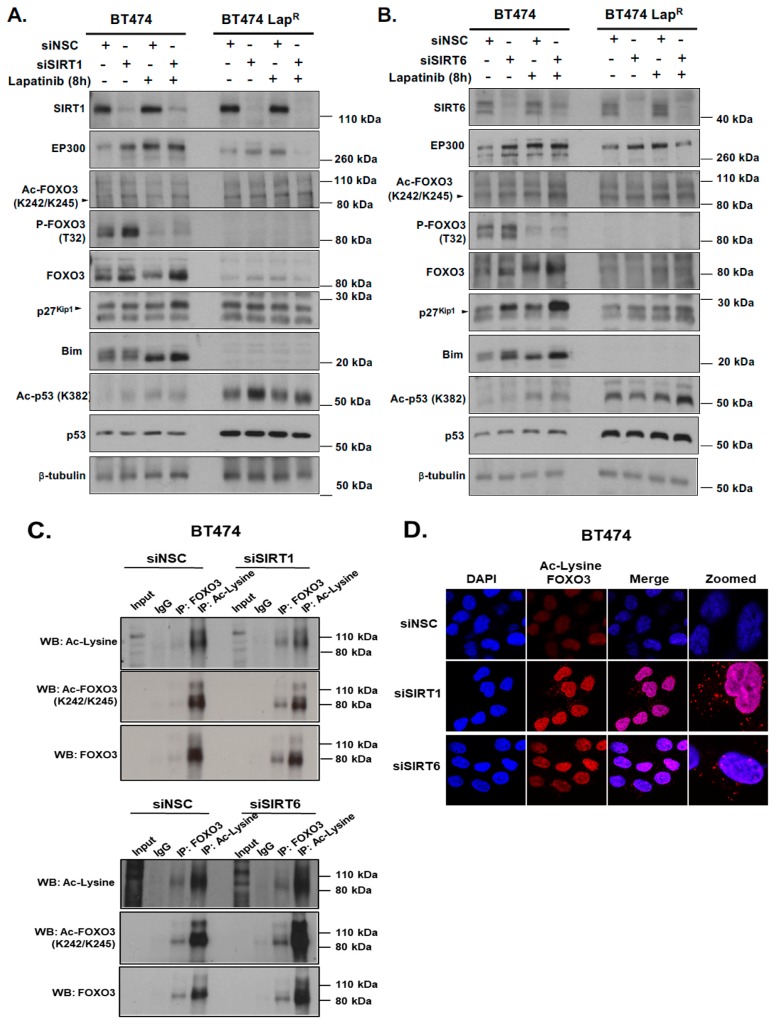
Silencing of SIRT1 and SIRT6 with or without Lapatinib treatment correlates with increased FOXO3 acetylation and its transcriptional activity. (**A**,**B**) BT474 and BT474 Lap^R^ cells were transfected with Control (siNSC), siSIRT1 or siSIRT6 siRNAs and treated with or without 1 µM Lapatinib for 8 h. After harvesting, protein lysates were obtained and subsequent western blotting was performed to assess FOXO3 acetylation and its transcriptional activity using antibodies against Ac-FOXO3, FOXO3, p27^Kip1^, Bim and others as indicated. The β-tubulin was used as a loading control. (**C**) Co-immunoprecipitation (Co-IP) was performed with anti-FOXO3 and anti-acetylated lysine antibody. Subsequent immunoblotting was performed using antibodies against Ac-Lys, Ac-FOXO3 and FOXO3. The anti-IgG antibody was used as a negative control. (**D**) BT474 and BT474 Lap^R^ cells were transfected with siNSC/siSIRT1/siSIRT6 siRNAs. After 24 h of transfection, cells were seeded in 8 chamber wells plate. Cells without Ac-Lys and FOXO3 antibodies were used as negative control. Representative confocal images are shown. Cells were visualized with a Leica TCS SP5 confocal microscope equipped with a 63× oil immersion objective and LAS-AF software. Each red spot represents for a single interaction and DNA was stained with 4,6-diamidino-2-phenyl indole (DAPI) in blue. Scale bar: 10 µm.

To further investigate the mechanisms regulating FOXO3 acetylation we determined how knocking-down deacetylases SIRT1 and SIRT6 affected FOXO3 activation following lapatinib treatment. The levels of acetylated FOXO3 increased after knocking down of SIRT1 and SIRT6, which in turn activated FOXO3 downstream anti-proliferative target gene expression such as p27^Kip1^ and Bim in BT474 cells (Figure 6A,C). Moreover, the expression of FOXO3, p27^Kip1^ and Bim were found to be also increased after lapatinib treatment upon sirtuin-depletion (Figure 6A,C) (Supplementary Figure S8F,G). In addition, EP300 expression was up-regulated upon SIRT1 or SIRT6 knock-down, that further increased in cells treated with 1 µM lapatinib for 8 h. The increased acetylation of FOXO3 upon lapatinib treatment and SIRT1 knockdown was also confirmed by co-immunoprecipitation experiments as FOXO3 or acetyl-Lys antibodies pulled down more complexes, detected with both total FOXO3 and acetylated FOXO3 (K242/245) antibodies, upon sirtuin RNA interference (Figure 6B).

The acetylation status of FOXO3 after knocking down of SIRT1 and SIRT6 was further examined in a more sensitive and efficient way by Duolink PLA using antibodies for FOXO3 and to acetylated Lys. Depletion of SIRT1 or SIRT6 induced the formation of red specks indicating FOXO3 acetylation in BT474 cells (Figure 6D). Collectively, these results suggest that SIRT1 and SIRT6 regulate the expression and the activity of FOXO3 and may have a role in fine-tuning FOXO3 acetylation.

### 2.8. Knockdown of SIRT6 Increases Lapatinib Cytotoxicity in Sensitive Cells

**Figure 7:**
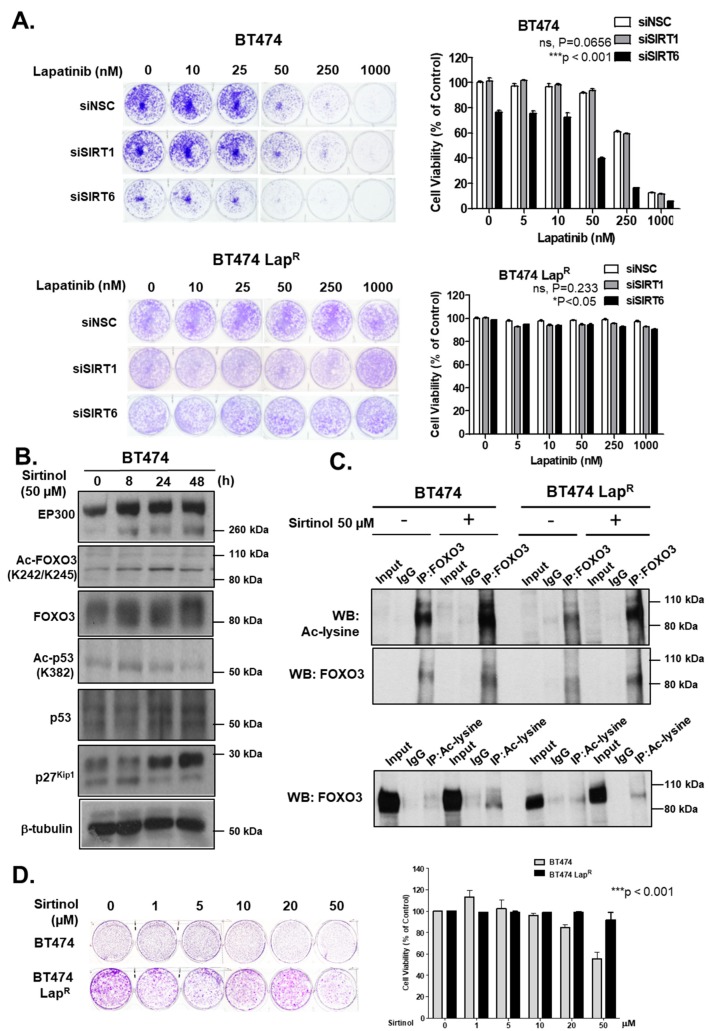
Silencing of SIRT6 as well as its inhibitor sirtinol increases long-term lapatinib cytotoxicity in BT474 cells. (**A**) BT474 and BT474 Lap^R^ cells were transfected with siNSC, siSIRT1 or siSIRT6 siRNA smartpool. After 24 h of transfection, 1000 cells were seeded per well, in 6-well plates. Cells were allowed to grow overnight and then treated with the lapatinib concentration indicated (0, 5, 10, 50, 250 and 1000 nM). At the end of this time period, cells were fixed with 4% para formaldehyde and stained with crystal violet. The stain was solubilized with 33% acetic acid and absorbance at 592 nm were obtained. Values obtained were converted to percentage of viability with non-silenced cells set to 100% viability. Data were finally normalized to the respective NSC transfected cells. (**B**) Protein lysates of BT474 cells obtained following treatment with or without 50 µM sirtinol for different time-point and were assessed by western blotting to check the expression of Ac-FOXO3, p27^kip1^ and for others indicated. (**C**) Co-immunoprecipitation (Co-IP) was performed with an anti-FOXO3 antibody. Subsequent immunoblotting was performed using antibodies against Ac-Lysine and FOXO3. A reverse Co-IP was performed with a pan acetylated lysine antibody and subsequent immunoblotting was done by using anti-FOXO3 antibody. The anti-IgG antibody was used as a negative control. (**D**) Clonogenic assay was employed after treating with different concentrations of sirtinol for 24 h and stained with crystal violet after 2–3 weeks. The stain was solubilized with 33% acetic acid and absorbance at 592 nm were measured.

To ascertain the role of SIRT1 and SIRT6 in long-term survival of cells upon lapatinib treatment, we performed clonogenic assays comparing the ability of BT474 and BT474-Lap^R^ cells to survive and form colonies upon RNA interference and in response to lapatinib treatment. Depletion of SIRT6 reduced cell proliferation in comparison with the siNSC control in the absence of lapatinib treatment (Figure 7A). Upon lapatinib treatment, SIRT6 knock-down BT474 cells showed greater sensitivity than siNSC control cells, especially at 50 nM or higher concentrations (Supplementary Figure S5). These effects were not observed upon SIRT1 depletion in BT474 cells. On the other hand, BT474-Lap^R^ cell lines did not show any significant changes in clonogenicity or lapatinib sensitivity upon SIRT-depletion (Figure 7A). Thus, depletion of SIRT6 but not SIRT1, increases lapatinib sensitivity in BT474 cells.

### 2.9. Inhibition of Sirtuins by Sirtinol Induces FOXO3 Acetylation and Lapatinib Sensitivity

Due to the role of sirtuins in lapatinib response described above, we determined the effect of a sirtuin inhibitor, sirtinol, in both sensitive and resistant BT474 cell lines. EP300 protein levels increased upon sirtinol treatment up to 48 h in BT474 cells (Figure 7B) (Supplementary Figure S8H). In addition, Ac-FOXO3 and Ac-p53 levels also increased, although with different kinetics. Ac-FOXO3 peaked after 24 h whereas Ac-p53 peaked after 8 h sirtinol treatment. Furthermore, sirtinol treatment increased total FOXO3 expression thus up-regulating its downstream target gene p27^Kip1^ expression in BT474 cells. To confirm the elevated acetylation status of FOXO3, we performed co-IP experiments. Subsequent western blotting result showed a significant increase of acetylation after sirtinol treatment for 24 h in BT474 cells (Figure 7C, upper panel) that was validated by reverse co-IP (Figure 7C, lower panel). Although there was also a slight increase in FOXO3 acetylation upon sirtinol treatment in lapatinib-resistant cells (Figure 7C, upper panel), this was not further confirmed in reverse co-IP experiments (Figure 7C, lower panel). Clonogenic assays confirmed that sirtinol inhibits proliferation at 20–50 µM in BT474 cells but not in lapatinib-resistant cells (Figure 7D). Collectively, these data indicate that sirtuins have an important role in FOXO3 acetylation, activation and lapatinib sensitivity in BT474 cells but not BT474-LapR cells.

### 2.10. FOXO3 Expression Correlates with SIRT6 and SIRT1 Levels in Different Breast Cancer Subtypes

**Figure 8:**
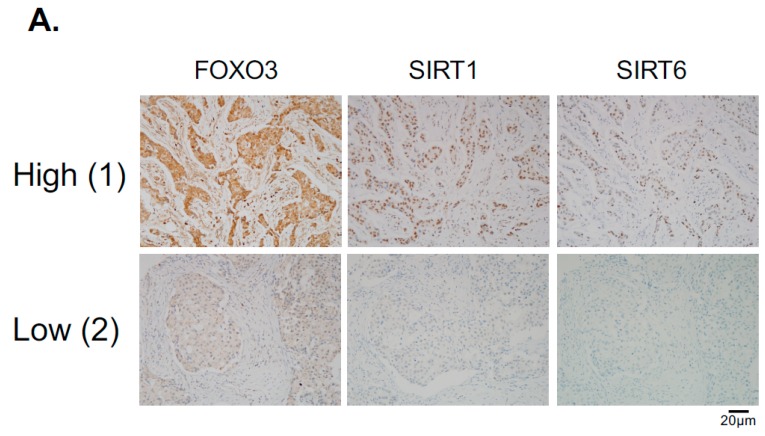

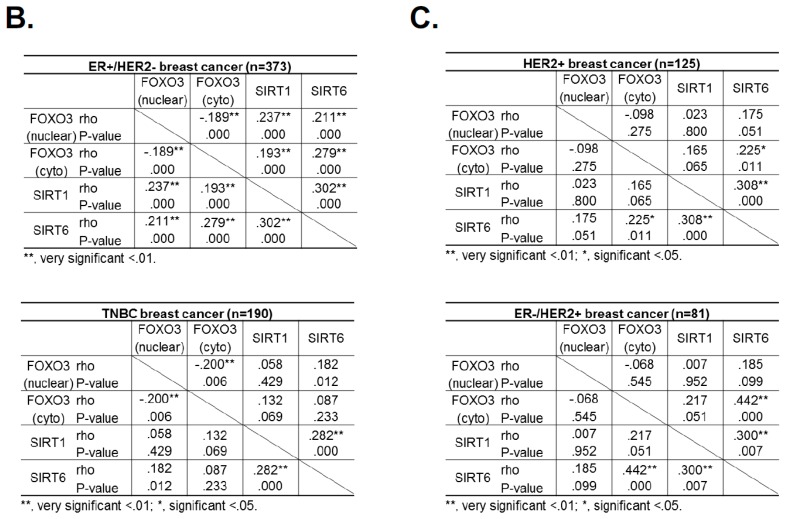
FOXO3 expression correlates with SIRT6 and SIRT1 levels in different breast cancer subtypes. (**A**) Representative immunohistochemical staining images from two samples (patient 1 and 2) showing correlations between FOXO3, SIRT1 and SIRT6 expression. Scale bar: 20 µm. (**B**,**C**) Staining results and Chi-square analysis. Chi-square statistical analysis was used to test the correlations between FOXO3, SIRT1 and SIRT6 expression in patients using SPSS 16.0. In statistical analysis, *p* < 0.05 was considered as significant (* *p* < 0.05, ** *p* < 0.01).

Hitherto, our data showed that SIRT1 and SIRT6 directly inhibit FOXO3 acetylation and its activity. To investigate this finding further, we studied the relationships between FOXO3, SIRT1 and SIRT6 expression by immunohistochemistry in a cohort of patients of different breast cancer subtypes (Figure 8A). The staining results showed that both cytoplasmic and nuclear FOXO3 was positively correlated with the expression levels of SIRT6, (*** *p* < 0.001) and SIRT1 (*** *p* < 0.001) in ER+HER2− breast cancer patients and cytoplasmic FOXO3 with SIRT6 (* *p* < 0.05) in triple-negative breast cancer (TNBC) samples (Figure 8B). Interestingly, cytoplasmic FOXO3 was found to be positively correlated with the expression levels of SIRT6 (*p* < 0.001) but not SIRT1 in HER2+ (ER+ and ER−) breast cancer patients (Figure 8C). However, SIRT1 demonstrated a potential positive trend with the expression levels of cytoplasmic FOXO3 in HER2+ breast cancer subtypes with negative estrogen receptor status (ER-) (Figure 8C). We and others have previously shown that constitutive high FOXO3 expression, particularly in the nucleus, predicts FOXO3 deregulation/inactivation and poor prognosis in patients [25,26]. Together these data provide further evidence that SIRT1 and SIRT6 cooperate to deregulate FOXO3 activity. As overexpression of the tumor suppressor FOXO3, specifically in the nucleus, can trigger cell cycle arrest and cell death, it is proposed that high levels of SIRT1/6 are expressed as an adaptive response to deacetylate FOXO3 and neutralize its potent antiproliferative function.

## 3. Discussion

Overexpression of *HER2* gene occurs in ~20–25% of primary breast cancers and is associated with poor clinical outcomes in the metastatic patients [27]. Monoclonal antibodies and tyrosine-kinase inhibitors are two major targeted therapies for HER2-positive breast cancer but these cancer cells often quickly develop adaptive responses to these HER2-targeted therapies [28]. Previously, we and others have shown FOXO3 to be an important regulator in the antiproliferative potency of the tyrosine kinase inhibitors Gefitinib and lapatinib [16,17,18,19,20]. To investigate further the role and regulation of FOXO3 in HER2-targeted therapies, a resistant derivative (BT474-Lap^R^) of the lapatinib-sensitive BT474 breast cancer cell line has been established. Lapatinib treatment causes dephosphorylation of EGFR, HER2 and AKT and subsequently dephosphorylation and activation of FOXO3. Moreover, EP300, FOXO3 and p27^Kip1^ expression is increased upon lapatinib treatment in sensitive BT474 cells but not in resistant BT474-Lap^R^ cells, suggesting that EP300-mediated acetylation increases FOXO3 activity and expression in response to lapatinib in sensitive but not the resistant BT474 cells. In addition, Duolink PLA assays confirm that lapatinib induces interactions between EP300 and FOXO3 and the associated FOXO3 acetylation. All together the results suggest that effective lapatinib response involves EP300 and FOXO3 interaction which results in FOXO3 acetylation and subsequent antiproliferative target gene expression in BT474 sensitive cells. Notably, this EP300-mediated FOXO3 acetylation requires the acetyl-transferase activity of EP300 as the acetyl-transferase deficient HAΤ Δ1472–1522 mutant fails to cause FOXO3 acetylation in BT474 cells.

To evaluate the effect of FOXO3 acetylation more thoroughly, transient EP300 over-expression has been performed in both BT474 and BT474-Lap^R^ cells. The results show that EP300 overexpression induces FOXO3 expression and acetylation, which promotes the expression of the anti-proliferative target genes, including *p27^Kip1^* and *Bim* in the sensitive BT474 cells. However, FOXO3 is deregulated and downstream targets uncoupled from FOXO3 in the resistant cells. In agreement, overexpression of FOXO3 increases both short and long-term lapatinib sensitivity in BT474 but not in BT474-Lap^R^ cells. ChIP assays provide further evidence that the insensitivity of the resistant cells to lapatinib treatment could be due to the failure of FOXO3 to be recruited to its target genes. Together, these results suggest a significant role of EP300-mediated acetylation of FOXO3 in regulating HER2-positive breast cancer cell survival and their lapatinib sensitivity.

Notably, consistent low levels of EP300 and high levels of nuclear sirtuins (SIRT1 and SIRT6) are found in BT474-Lap^R^ cells. This suggests that the de-acetylation of FOXO3 by these nuclear SIRTs might also contribute to lapatinib resistance. Therefore, FOXO3 acetylation, regulated coordinately by EP300 and sirtuins, is an important PTM which regulates FOXO3 expression and transcriptional activity to modulate lapatinib sensitivity in breast cancer (Supplementary Figure S6). Consistently, sirtuin-mediated deacetylation of FOXO3 has been reported to restrict EP300-mediated transactivation of FOXO3 and promote stress resistance and longevity [2,8]. Our data show that SIRT1 and SIRT6 knockdown in combination with lapatinib treatment causes an upregulation of FOXO3 expression and acetylation. Enhanced FOXO3 acetylation mediated by SIRT-depletion in BT474 cells also induces the expression of FOXO3 downstream target genes, such as *p27^Kip1^* and *Bim*. Moreover, knockdown of SIRT6 reduces the cell proliferation and increases lapatinib sensitivity in BT474 cells, although silencing of SIRT1 does not result in any significant effects on drug sensitivity. This could be due to the fact that SIRT6 plays a more significant role in the inhibition of FOXO3 acetylation in these cells and/or that SIRT6 can functionally compensate for the loss of SIRT1. In agreement, the pan-sirtuin inhibitor, sirtinol which can target both SIRT1 and SIRT6 increases p27^Kip1^ expression in the BT474 cells.

Since the levels of SIRT1 and SIRT6 do not change in response to lapatinib treatment, they are unlikely to have a direct role in modulating lapatinib signaling but may instead play a part in fine-tuning FOXO3 acetylation and therefore lapatinib sensitivity by setting the baseline for the de-acetylation activity in both lapatinib sensitive and resistant cancer cells. The finding that inhibition of SIRT1/6 by sirtinol does not increase lapatinib sensitivity in the resistant cells is likely due to the fact that EP300, which plays a critical part in FOXO3 acetylation, is not induced in the lapatinib resistant cells. This further suggests that SIRT1 and SIRT6 cooperate to set a threshold for FOXO3 acetylation in the cancer cells. Immunohistochemical staining results also show that FOXO3 expression correlates positively with SIRT6/1 levels in different breast cancer subtypes. As high FOXO3 expression, particularly in the nucleus, predicts FOXO3 deregulation/inactivation and poor prognosis in patients [25,26], these IHC results provide further evidence that SIRT1 and SIRT6 cooperate to deregulate FOXO3 activity.

Lapatinib treatment is correlated with EP300 expression, FOXO3 acetylation and dephosphorylation (T32) and an increase in FOXO3 expression as well as cytoplasm to nucleus translocation in the sensitive BT474 cells. This suggests that lapatinib-induced FOXO3 acetylation via EP300 might drive FOXO3 dephosphorylation (T32), nuclear translocation and activation. Consistently, overexpression of wild-type EP300 alone can also induce FOXO3 acetylation, dephosphorylation (T32) and transcriptional activation in the sensitive BT474 cells. Interestingly, EP300 has previously been shown to mediate FOXO3 acetylation to antagonize its antiproliferative activity through restricting Bim expression [29]. In contrast, we show in here that EP300 promotes the antiproliferative function of FOXO3 through mediating its acetylation. In agreement with our findings, FOXO3 acetylation has been shown to mediate the antiproliferative functions of glucocorticoid in B acute lymphoblastic leukemia (B-ALL) [30]. Notably, we found that both HER2 and P-AKT are downregulated while EGFR is upregulated in the Lapatinib resistant cells and this is at odds with a previous study showing that both HER2 and P-AKT were upregulated in the Lapatinib resistant cells [1]. The reason for the discrepancy is unclear. However, the HER2-inhibitor resistant cells used in the other study were primarily generated by retrovirally transducing hotspot PIK3CA mutations into HER2-amplified cells [31]. The transduced PIK3CA mutations would maintain the high HER2 expression and high P-Akt activities as the PIK3CA mutations function downstream of HER2 will directly activate AKT activity. We have previously found that FOXO3 can control Akt activity (P-Akt) in a negative feedback mechanism [32]. The Lapatinib-resistant cells have low FOXO3 expression as an adaptive response during the development of lapatinib resistance and as a result Akt activity (P-Akt) is also lower in the resistant cells.

In summary, in the present study we identify a role of EP300-mediated FOXO3 acetylation in the regulation of lapatinib sensitivity in breast cancer. Our findings also suggest that SIRT1 and SIRT6 also play a coordinate role in fine-tuning FOXO3 acetylation and therefore lapatinib sensitivity by setting a threshold for the de-acetylation activity in both lapatinib sensitive and resistant cancer cells. Our data suggest that FOXO3 acetylation by EP300 could be a potential marker for the identification of tumors that are likely to be sensitive to these drugs and indicate a novel therapeutic strategy to overcome lapatinib resistance in HER2-positive breast cancers.

## 4. Materials and Methods

### 4.1. Cell Lines, Cell Culture and Treatments

The BT474 cell line was originally obtained from the American Type Culture Collection (ATCC). Human breast cancer cell line, MCF-7 (Michigan Cancer Foundation-7) was acquired from the Cell Culture Service of Cancer Research UK, where they were tested and authenticated. lapatinib resistant BT474 cells (denoted as BT474 Lap^R^) were derived from parental BT474 cells that were continuously exposed to 1 µM lapatinib for over 3 months. BT474-LapR cells were routinely maintained with 1 µM lapatinib in the culture medium. Wild type mouse embryonic fibroblasts (MEFs) and triple knock-out *foxo1/3/4^-/-^* MEFs were kind gifts from Prof. Boudewijn Burgering, UMC, Utrecht, the Netherlands and have been described previously [33]. All cells were cultured in Dulbecco’s modified eagle’s medium (DMEM) (Sigma Aldrich, Poole, UK) and supplemented with 10% (*v*/*v*) fetal calf serum (FCS) (First Link Ltd., Birmingham, UK), 100 Unit/mL penicillin/streptomycin (Sigma-Aldrich, UK) and 2 mM glutamine and maintained at 37 °C in a humidified atmosphere containing 10% CO_2_. Cultured cells were also maintained by serial passaging using 25 g/L trypsin solution (Sigma Aldrich, Poole, UK) in 0.02% ethylene diamine tetra acetic acid (EDTA) (Sigma Aldrich, Poole, UK) when 70–80% confluence was reached.

### 4.2. Plasmids

Plasmid pcDNA3.1-p300 (plasmid #23252) and the acetyl-transferase mutant pcDNA3.1-p300 (HAT-) (plasmid #23254) were obtained from Addgene (Addgene, MA, USA). Empty vector pcDNA3.1 was purchased from Invitrogen. All plasmids were cloned into *E. coli* DH5α and the Purelink^®^ HiPure Plasmid Maxiprep Kit (Thermo Fisher Scientific, Hemel Hampstead, UK) was used to isolate plasmid DNA as indicated in the manufacturer’s instructions. This kit uses an alkaline lysis protocol to isolate plasmid DNA that is bound to a positively charged resin before elution under high salt conditions. For gene silencing.

### 4.3. Breast Cancer Cell Transfections

BT474 and BT474-Lap^R^ cell lines were seeded to achieve approximately 60–70% confluency in T75 flasks before transfection. The plasmid-encoding human EP300, pcDNA3.1-EP300, the acetyl-transferase mutant, pcDNA3.1-p300 (HAT-) and the empty vector were transfected into cells using XtremeGene (Sigma-Aldrich). Transfection was performed at a 3:1 XtremeGene: DNA ratio using 8 µg of plasmid following manufacturer’s instruction. Transfected cells were counted and used for any subsequent assays 24 h post-transfection. For gene silencing, cells were plated in at 60–70% densities. The following day, cells were transfected with ON-TARGET plus siRNAs (GE Dharmacon, Horizon Discovery LTD, Cambridge, United Kingdom) targeting EP300 (L-003486-00-0005), SIRT1 (L-003540-00-0005) or SIRT6 (L-013306-00-0005) using oligofectamine (Invitrogen, Thermo Fisher Scientific, UK) according to the manufacturer’s protocol. Non-Targeting siRNA pool (GE Dharmacon; D-001210-01-05) was used as transfection control.

### 4.4. Duolink In Situ PLA In Situ

The analysis was carried out according to the manufacturer’s instructions [34]. This assay is used to detect, visualize and quantify protein expression, protein interactions and specific post-translational protein modification [34]. In the cases of detecting protein interactions and post-translational modification, two primary antibodies are required which are raised from different species. The secondary antibodies called PLA probes (PLA probe MINUS and PLA probe PLUS) and each one is conjugated with a unique oligonucleotide, were obtained from Sigma-Aldrich (DUO92008 SIGMA; POOLE UK). When the two PLA probes are in close proximity (<40 nm), the oligonucleotides will hybridize and join to a closed circle by adding enzymatic ligation solution. The ligated circle as a template, is then amplified through rolling circle amplification and generating several-hundredfold repeated sequence product subsequently. Since the amplification solution consists of nucleotides, polymerase as well as fluorescently labelled oligonucleotides, the replicated product can be easily visible as distinct fluorescent spot when viewed with a fluorescence microscopy.

### 4.5. Protein Extraction and Quantification

Whole cell extracts were prepared by harvesting cells by using 1×trypin-EDTA. Once detached, medium was added to inactivate the trypsin and samples were spun in 2000× rpm for 5 min. The supernatant was discarded and cell pellets were washed in PBS and spun for an additional 5 min (1200× *g*). The supernatant was discarded and the cell pellet was frozen at −80 °C until lysis was performed. Frozen pellets were lysed in lysis buffer (150 mM NaCl, 50 mM Tris-HCl (pH 7.4), 5 mM EDTA, 5 mM Dithiothreitol (DTT), 1% NP-40 (or IGEPAL) and 1 tablet of protease inhibitors (“Complete” protease inhibitor mixture, as instructed by the manufacturer, Roche Applied Science; Burgess Hill, UK) supplemented with phosphatase inhibitors (1 mM sodium fluoride (NaF), 1 mM sodium orthovanadate (Na_3_VO_4_) and 1 mM PMSF as protease inhibitor. The samples were kept on ice for 10 min, vortexing every 3 min to disrupt the cell membranes. The supernatant (protein extract) was finally collected by centrifugation at 800× *g* for 10 min at 4 °C. Protein concentration was determined using the Pierce BCA Protein Assay Reagents A and B (Thermo Scientific) according to manufacturer’s instruction. Absorbance was read at 562 nm using the Sunrise spectrophotometer (Tecan; Reading, UK). Protein concentrations were determined by the equation of absorbance × 25 = µg/µL.

### 4.6. Western Blotting

SDS-PAGE gels are consisting of an upper stacking and a lower resolving gel. The resolving gel was made with varying amounts of bis-acrylamide solution, according to the molecular size of the protein being isolated with percentages of gels used varying from 7 to 14. Lower percentage gels allowed better examination of proteins of higher molecular weight (MW) and vice versa. The bis-acrylamide with 25% ammonium persulfate (APS) and tetramethylethylenediamine (TEMED) were used as the catalysts for gel polymerization.

To separate proteins, 20 μg of protein lysate was added with 2 × SDS loading buffer (200 mM Tris-HCl pH 6.8, 6% sodium dodecyl sulfate (SDS), 2 mM EDTA, 10% 2-mercaptoethanol, 10% glycerol, 0.02% bromophenol blue) and boiled for 5 min at 100 °C. Samples were then spun at 800× *g* for 1 min and the appropriate volume of sample was loaded into the stacking wells. SDS-PAGE gels were run in running buffer (25 mM Tris-Base, 250 mM glycine, 0.1% (*w*/*v*) SDS) at 60 V through the stacking gel and then 100 V through the resolving gel.

Once the proteins had been separated by SDS-PAGE, proteins were electro-transferred on to 0.45 μm Protran nitrocellulose membranes (Schleicher and Schuell, Whatman, Brentford, UK) using a wet tank blotting system (Bio-Rad Laboratories Trans-Blot Cell) in transfer buffer (25 mM Tris, 190 mM glycine and 20% (*v*/*v*) ethanol) for 90 min at 90 V. Tanks were packed in ice to avoid overheating.

Membranes were then blocked in 5% (*w*/*v*) bovine serum albumin (BSA) in Tris-buffered solution with 0.05% Tween-20 (TBST, pH 7.5) for 1 h at room temperature. Primary antibody incubation was performed with antibodies diluted in a 5% BSA-TBST solution overnight at 4 °C. The membranes were washed four times with TBST, every 5 min with 50 mL TBST at room temperature. Membranes were then incubated in their respective secondary antibodies (anti-rabbit or anti-mouse; Santa-Cruz Biotechnologies; Santa Cruz, CA, USA) coupled with horseradish peroxidase (Dako, Ely, UK) at a 1:2000 dilution for 1 h at room temperature. After that, membranes were washed for five times in every 5 min with 50 mL TBST at room temperature to remove excess secondary antibodies. Proteins were then visualized using enhanced chemiluminescence detection system (GE Healthcare, Cambridge, United Kingdom).

Primary antibody against Ac-FOXO3 (K242/K245) developed by Professor Eric lam and produced in PickCell Laboratories. Other primary antibodies used were P-EGFR (Cell signaling, #2234), EGFR (Cell signaling, #4267), P-HER2 (Cell signaling, #2244), HER2 (Cell signaling, #2242), P-AKT (S473) (Cell signaling, #4060), AKT (Cell signaling, #4685), β-tubulin (Santa Cruz Biotechnology, sc9104), EP300 (Abcam, ab10485), P-FOXO3 (T32) (Cell signaling, #9464), FOXO3 (cell signaling, #2497), Ac-p53 (K382) (Cell signaling, #2525), p53 (Santa Cruz Biotechnology, #sc98), FOXM1 (Santa Cruz Biotechnology, sc502), p27^Kip1^ (Santa Cruz biotechnology, sc-528), SIRT1 (Abcam, Ab32441), SIRT2 (Santa Cruz Biotechnology, sc20966), SIRT6 (Cell Signaling, 2590 and Abcam, ab62739), HER3 (Cell signaling #4754), H3K27Ac (Abcam, #ab4729), Bim (Cell signaling, #2933), p130 (BD Biosciences, #610272), p16^INK4a^ (BD Biosciences, #554079), Acetylated lysine (Cell signaling, #9441). The original blots (Supplementary Figure S7) were scanned and densitometry readings/intensity ratio of each band calculated for analysis (Supplementary Figure S8).

### 4.7. Real-Time Quantitative PCR

Cells were harvested by centrifugation (1200× *g*) and cell pellets were stored at −80° until RNA extraction. Total mRNA was obtained from frozen cell pellets using the RNeasy Mini Kit (Qiagen, Manchester, United Kingdom) according to manufacturer’s instructions. mRNA purity and concentration were determined by measuring the spectrophotometric absorption at 260 nm and 280 nm on NanoDrop ND-1000.

Two µg of extracted total RNA was used as a template for first strand cDNA synthesis reaction and the resulting first-strand cDNA was used as template in the real-time PCR. The initial reaction mix to reverse transcribe the total RNA contains random primers, 10 µM of each dNTP and Superscript III reverse transcriptase (Invitrogen). The initial mixture was heated for 65 °C (5 min) for denaturation and cooled on ice. 5× First-Strand Buffer, 0.1 M DDT and RNaseOUT RNase Inhibitor (40 units/µL) were added to the denatured samples and were returned to the PCR machine programmed to incubate at 25 °C (5 min), 50 °C (60 min) for annealing and a final deactivation by heating at 70 °C for (15 min). By pooling equal amounts of all diluted cDNA samples, a cDNA standard was obtained. Four serial 1:4 dilutions (1/4, 1/16, 1/64 and 1/256) of the standard in water were used for generation of standard curve points. The nonregulated ribosomal housekeeping gene *L19* was used as an internal control to normalize gene expression between samples. All amplifications were performed in triplicate.

Real time quantitative PCR (RT-qPCR) was performed using 100 ng cDNA which was added to SYBR-Green Master Mix (Applied BioSystems). All RT-qPCR assays were assayed in 96-well plates in the ABI PRISM^®^ 7900 HT Fast Real-time PCR System (Applied BioSystems) on a cycling program of 90 °C for 10 min for enzyme activation followed by 40 cycles of denaturation and primer annealing/extension consisting of 95 °C for 10 s and 60 °C for 30 s, respectively.

Primers used for RT-qPCR are summarized in Table 1.

### 4.8. Co-Immunoprecipitation Assay (Co-IP)

BT474 and BT474-Lap^R^ cells were harvested as already described for protein extraction. Cell pellets were lysed in IP lysis buffer [1% Nonidet P-40, 150 mM NaCl, 50 mM Tris-HCl (pH 7.4), 5 mM EDTA) supplemented with fresh 1 mM PMSF, 1 mM NaF, 1 mM Na_3_VO_4_, 1 mM DTT and one EDTA-free protease inhibitor tablet (Complete protease inhibitor cocktail; Roche, Lewes, UK). The samples were incubated for 20 min on ice, vortexing every 5 min to disrupt cell membrane and then centrifuged at 800× *g* for 10 min at 4 °C to collect protein extract in new Eppendorf tube. After protein quantification using the BCA Protein Assay Kit (Pierce, Rockford, IL, USA), 10% of the total protein (25–50 µg) from each sample was kept as an input control. Subsequently, 250–500 µg of each lysate was rotated with 20 µL of pre-washed protein agarose Dynabeads A (Novex, Life |Technologies, Carlsbad, CA, USA) for 4 h at 4 °C in order to reduce non-specific binding. After the pre-clearing step, the lysates were transferred to new Eppendorf tubes containing new beads that have been previously washed three times with IP lysis buffer. Samples were incubated overnight with 0.5 µg of primary antibody-FOXO3a (sc-11351, Santa Cruz), Anti-acetyl lysine (Cell-Signaling technology) or IgG negative control (2729, Cell-Signaling technology) at 4 °C on a rotator. The supernatant was discarded on the following day and beads were washed three times with IP lysis buffer. Samples were boiled with 2 × SDS sample buffer for 5 min at 100 °C and were analyzed by SDS-PAGE followed by western blotting.

### 4.9. Chromatin Immunoprecipitation (ChIP)

BT474 cells were either left untreated or treated with 1 μM Lapatinib for 2 and 4 h, before harvesting. DNA fragments were purified using phenol chloroform (Sigma Aldrich, St Louis, MO, USA). For subsequent qPCR analysis, the 7900HT Fast Real-Time PCR System (Applied Biosystems, Carlsbad, CA, USA) was used. The first stage was held at 95 °C for 10 min and subsequent amplification was carried out by 40 cycles of the following three steps: 95 °C for 15 s, 60 °C for 30 s and 72 °C for 30 s. A dissociation stage consisting of three steps (95 °C, 60 °C and 95 °C for 15 s each) was also performed to test primer specificity. The fold enrichment method was finally used for analysis the immunoprecipitated DNA, with each ChIP signal normalized to its respective DNA concentration, input sample and background signal obtained for the IgG negative control. Primer pair sequences for ChIP analyses are

Rictor-F: AAACAAACCCATCTCCACTGC;

Rictor-R: TTTCCCTTCTGCTTCCCTTT;

p27^Kip1^-F: CCGTTTGGCTAGTTTGTTTGTCT;

p27^kip1^-R: TGACTGCTGGAGGGGTACTG;

RAD51-F: ATGCGAGTAGGAGGCTCAGA;

RAD51-R: AGCGCTCTTGTGGTTTGTTT;

### 4.10. Sulphorhodamine B (SRB) Colourimetric Assay

Drug sensitivity to Lapatinib was tested using the SRB colorimetric assay. Drug sensitive and resistant BT474 cells were seeded 24-h post-transfection in 96-well plates, 3000 and 1000 cells per well, respectively. Cells were allowed to attach overnight and then treated with the Lapatinib concentration indicated (0.1, 1.0 or 1.5 µM). Cells were then fixed 24, 48 or 72 h following treatment using ice cold 20% (*w*/*v*) trichloroacetic acid (TCA) for 1 h. Cells were then washed, allowed to dry overnight and stained with 0.4% SRB in 0.1% (*v*/*v*) acetic acid for 30 min. Excess dye was washed off with 1% (*v*/*v*) acetic acid and 10 mM Tris was then used to dissolve SRB by shaking for 30 min at room temperature. Absorbance was finally measured at 492 nm using a Sunrise microplate reader (Tecan, Männedorf, Switzerland).

### 4.11. Clonogenic Assay

Long term sensitivity to Lapatinib was tested by the clonogenic assay. One day post-transfection, the cells were counted and plated into 6-well plates at a density of 1000 and 500 cells per well for drug sensitive and resistant BT474 cells, respectively. Colony formation was allowed for 28 and 14 days in sensitive and resistant BT474 cells, respectively. At the end of this period, cells were fixed with 1% Formaldehyde in PBS, washed three times with PBS and stained with 0.5% (*w*/*v*) crystal violet. Pictures were taken and the stain was solubilized with 33% acetic acid by shaking at room temperature until completely dissolved. Absorbance was finally measured at 592 nm using a Sunrise microplate reader (Tecan, Männedorf, Switzerland).

### 4.12. Patients and Tissue Specimens

The patient tissue samples came from a study consisting of 688 breast cancer patients who underwent surgery for primary breast cancer between 1993 and 1998, at the Department of Pathology, Asan Medical Center, Seoul, Korea. Formalin-fixed, paraffin-embedded tissue samples from these preoperatively chemo- and radiotherapy naive patients were available for analysis as previously described [35]. The expression levels of standard biomarkers, including estrogen receptor (ER), progesterone receptor (PR) and HER2, were reviewed by immunohistochemical staining at the time of diagnosis, independently by two pathologists. HER2-overexpressing tumors were defined as those that score of 3+ by immunohistochemistry (IHC) or by gene amplification using either fluorescence in situ hybridization or silver in situ hybridization [36]. Exemption from informed consent after the de-identification of information was approved by the Institutional Review Board of Asan Medical Center (2013-0641). Tissue microarray sections were evaluated with an automated immunohistochemical staining device (Benchmark XT; Ventana Medical Systems, Tucson, AZ, USA). Antibodies to FOXO3 (1:400 dilution; EMD Millipore, Billerica, MA, USA), SIRT1 (1:50 dilution; Abcam, Cambridge, MA, USA), SIRT6 (1:50 dilution; Cell Signaling Technology, Danvers, MA, USA) were used. Each case was evaluated by estimating the percentage and staining intensity (negative, 0; weak, 1; moderate, 2; and strong, 3) of tumor cells showing a cytoplasmic or nuclear staining pattern for FOXO3. We then classified the expression levels as high or low based on the mean staining intensity value obtained by the multiplication of each protein.

### 4.13. Statistical Analysis

Chi-square statistical analysis were used to test the correlations between protein expression of breast cancer patients, respectively using SPSS 16.0 (Imperial College London, Software Shop, UK); * *p* ≤ 0.05 and ** *p* ≤ 0.01 were considered as statistically significant and very significant, respectively. Other results shown represent the average of 3 independent experiments, which were each performed in triplicate (presented as the mean ± SEM). GraphPad Prism was used for statistical analysis (version 5, San Diego, CA, USA) and two-tailed Student’s *t*-test was used to compare the means. Differences between the two groups were considered statistically significant at *p* < 0.05. For comparisons between groups of more than two unpaired values, one-way analysis of variance (ANOVA) was used. Two-way ANOVA was used between groups of 2 variables and *p* < 0.05 was considered as statistically significant.

## Figures and Tables

**Table 1:** Forward and reverse primer sequences used for RT qPCR.

Gene	Primer Sequence (5′-3′)
EP300 forward	AAAAATAAGAGCAGCCTGAG
EP300 reverse	AGACCTCTTTATGCTTCTTCC
FOXO3 forward	TCTACGAGTGGATGGTGCGTT
FOXO3 reverse	CGACTATGCAGTGACAGGTTGTG
p27Kip1 forward	GAATAAGGAAGCGACCTGCAA
p27Kip1 reverse	TCTTCTGTTCTGTTGGCTCTTTTGT
RPL19 forward	GCGGAAGGGTACAGCCAAT
RPL19 reverse	GCAGCCGGCGCAAA
FOXM1 forward	TGCAGCTAGGGATGTGAATCTTC
FOXM1 reverse	GGAGCCCAGTCCATCAGAACT

## Supplementary Materials

The following are available online at https://www.mdpi.com/2072-6694/11/8/1067/s1, Figure S1: Validation of Lapatinib resistance in BT474 LapR cells, Figure S2A: Overexpression of FOXO3 in BT474 Lap^R^ cells, Figure S2B: Overexpression of FOXO3 in MEFs cells, Figure S3: Overexpression of EP300 or EP300 DHAT does not affect Lapatinib resistance in BT474 cells, Figure S4A: Protein expression analysis of SKBR3 and SKBR3 LapR upon lapatinib treatment, Figure S4B: EP300 overexpression enhances lapatinib sensitivity of SKBR3 cells, Figure S5: Silencing of SIRT6 increases lapatinib cytotoxicity in BT474 cells, Figure S6: Schematic model showing the regulation of FOXO3 acetylation by EP300 and SIRT1/6 and its subsequent outcome on Lapatinib sensitivity and resistance in breast cancer cells, Figure S7: Original full-length blots for the cropped/composite western blot images, Figure S8: Protein quantification using ImageJ from all the cropped/composite western blot images.
