# Supplementary material for: EP300 and SIRT1/6 Co-Regulate Lapatinib Sensitivity Via Modulating FOXO3-Acetylation and Activity in Breast Cancer
Source: Cancers (Basel). 2019 Jul 28;11(8):1067. doi: 10.3390/cancers11081067 (PMC6721388; doi:10.3390/cancers11081067)
Supplement: Supplementary file 1 [file cancers-11-01067-s001.zip › cancers-532228-supplementary/Supplementary Figure S1-8/Supplementary Fig S1.pdf]

Supplementary Figure S1

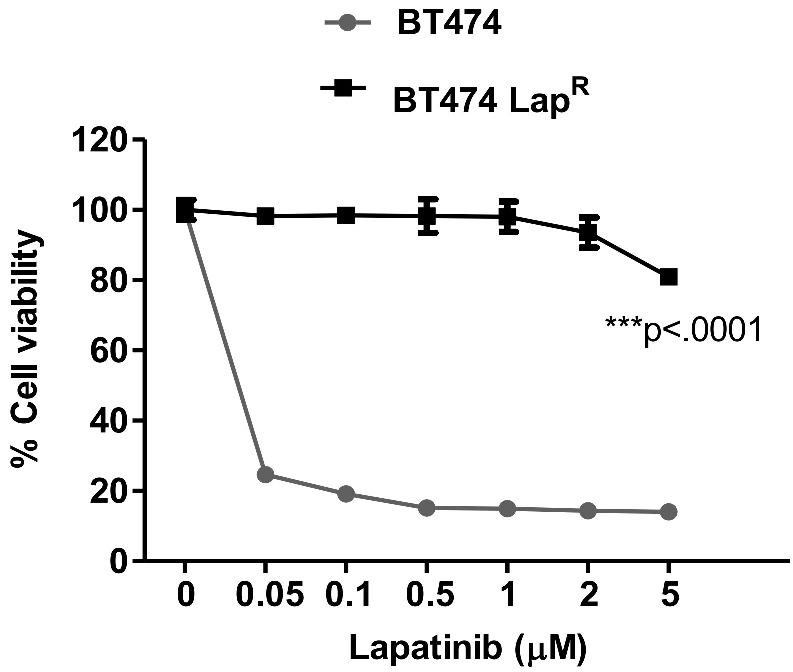

Supplementary figure S1. Validation of Lapatinib resistance in BT474 Lap^R cells
BT474 and BT474 Lap^R cells were seeded in 96-well plates and treated with lapatinib at a range of concentration from 0.05 to 5 μM. Twenty-four hours after treatment, cells were fixed and stained with the protein-binding dye SRB. Values obtained were normalised against the corresponding untreated controls and presented as percentages (The cell survival value was normalized to 100% both untreated cells). Bars represent the mean ± SEM of three independent transfection experiments (n=3, R=3) and statistical analysis was performed using 2-way ANOVA (***$p<0.001$, significant),
