# Supplementary material for: EP300 and SIRT1/6 Co-Regulate Lapatinib Sensitivity Via Modulating FOXO3-Acetylation and Activity in Breast Cancer
Source: Cancers (Basel). 2019 Jul 28;11(8):1067. doi: 10.3390/cancers11081067 (PMC6721388; doi:10.3390/cancers11081067)
Supplement: Supplementary file 1 [file cancers-11-01067-s001.zip › cancers-532228-supplementary/Supplementary Figure S1-8/Supplementary Fig S2.pdf]

Supplementary Fig. S2A

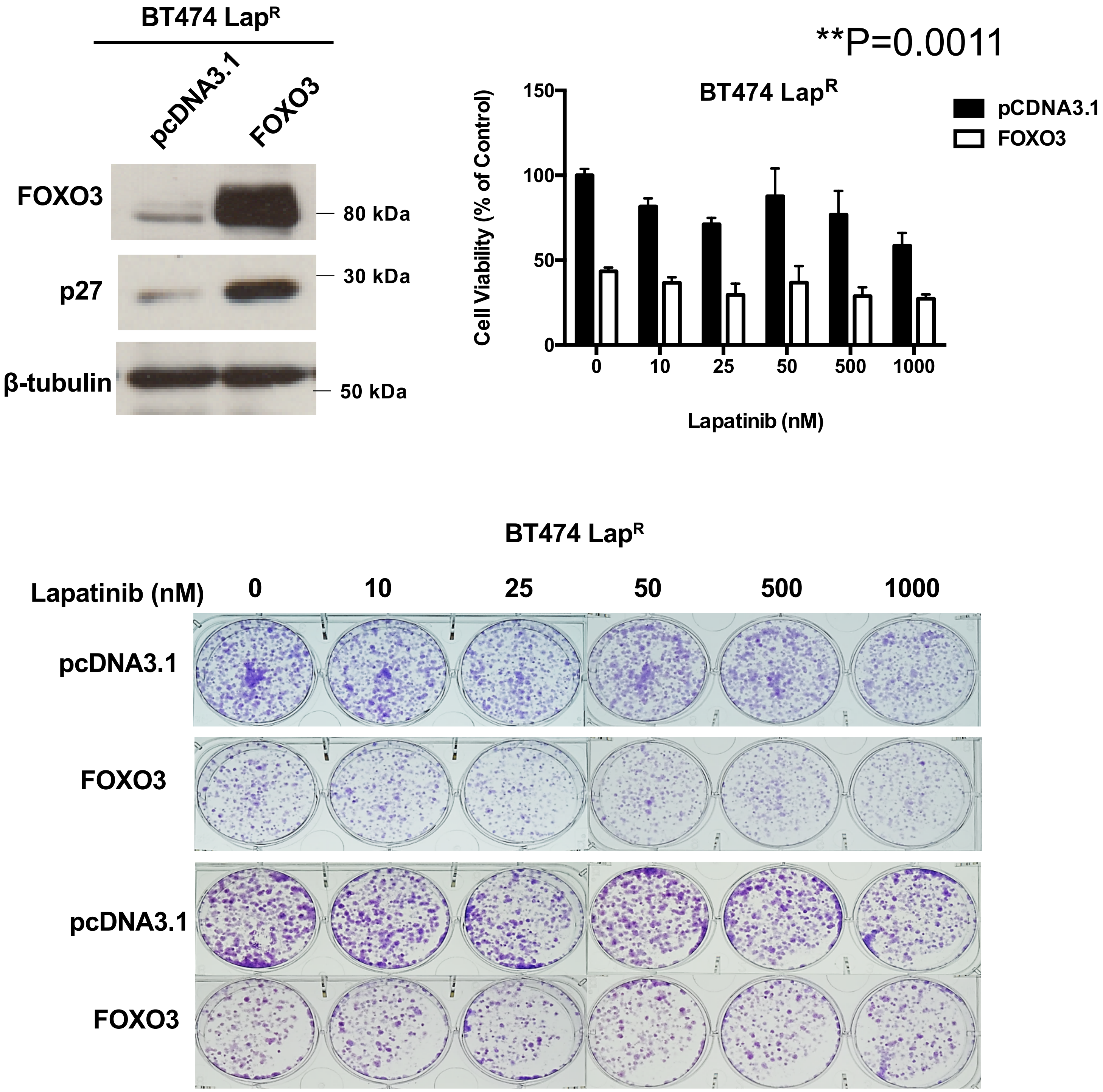

Supplementary Fig. S2A

BT474 Lap^R cells were transiently transfected with the empty vector pcDNA3.1 and the plasmid encoding FOXO3. The transfected cells were then seeded in 6 well plates (1000 cells per well), allowed to grow overnight and then treated with the lapatinib concentrations indicated (0,10, 25, 50, 500 and 1000 nM). Twenty-four hours following treatment the medium was changed and colony formation was allowed for 14 days. Cells were then fixed with 4% para formaldehyde and stained with crystal violet. The stain was solubilised with 33% acetic acid and absorbance obtained at 592 nm. Bars represent the mean ± SEM of three independent transfection experiments (n=3, R=3) and statistical analysis was performed using ANOVA analysis (*p<0.05, **p<0.01, ***p<0.001). Western blot analysis demonstrated FOXO3 overexpression.

Supplementary Fig. S2B

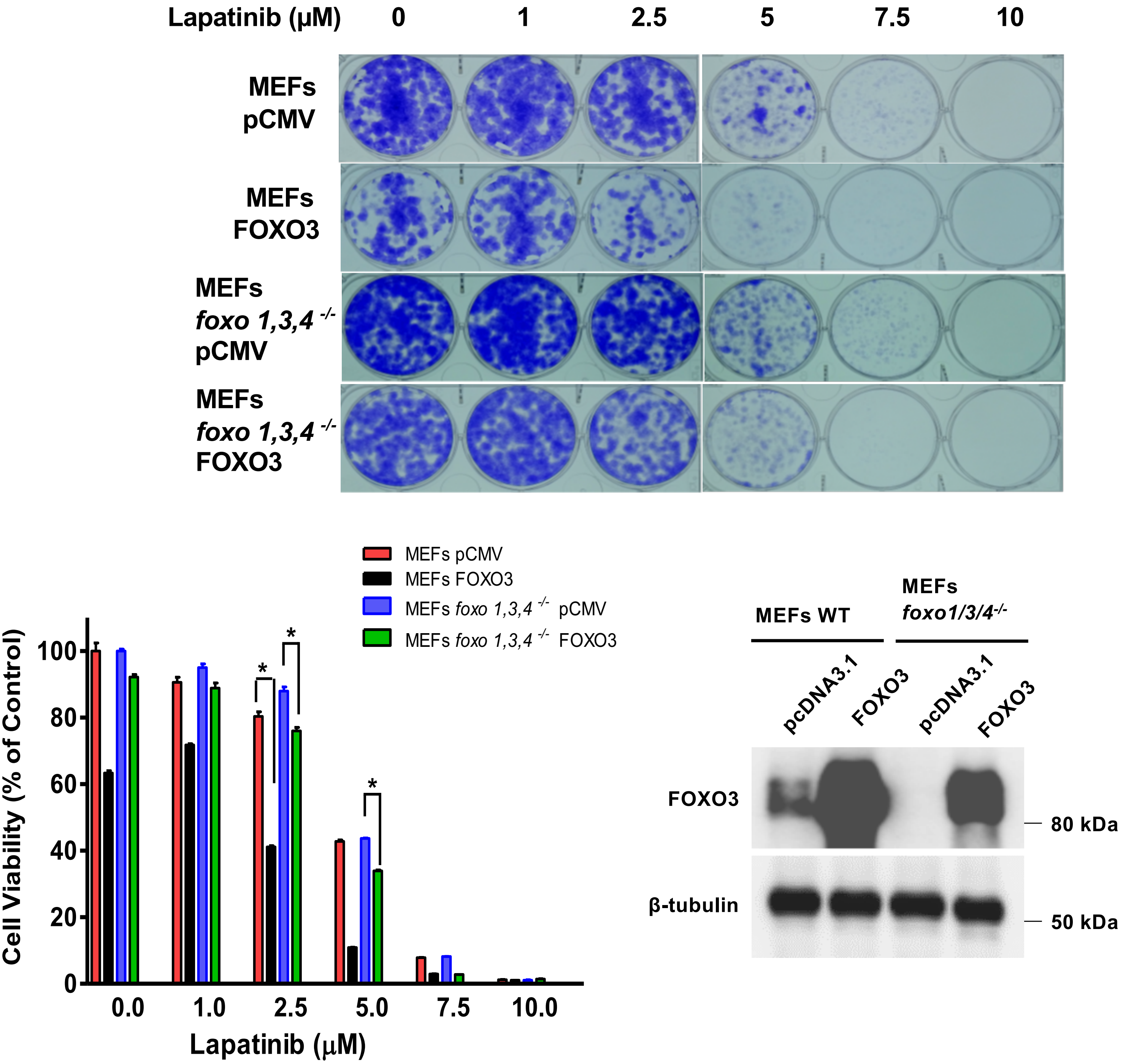

Supplementary Fig. S2B
Wild-type MEFs and *Foxo1,3,4*$^{-/-}$ MEFs cells were transiently transfected with the empty vector pcDNA3.1 and the plasmid encoding FOXO3. The transfected cells were then seeded in 6 well plates (1000 cells per well), allowed to grow overnight and then treated with the lapatinib concentrations indicated. Twenty-four hours following treatment the medium was changed and colony formation was allowed for 14 days. Cells were then fixed with 4% para formaldehyde and stained with crystal violet. The stain was solubilised with 33% acetic acid and absorbance obtained at 592 nm. Bars represent the mean ± SEM of three independent transfection experiments (n=3, R=3) and statistical analysis was performed using ANOVA analysis (*p<0.05, **p<0.01, ***p<0.001). Western blot analysis demonstrated FOXO3 overexpression.
