# Supplementary material for: EP300 and SIRT1/6 Co-Regulate Lapatinib Sensitivity Via Modulating FOXO3-Acetylation and Activity in Breast Cancer
Source: Cancers (Basel). 2019 Jul 28;11(8):1067. doi: 10.3390/cancers11081067 (PMC6721388; doi:10.3390/cancers11081067)
Supplement: Supplementary file 1 [file cancers-11-01067-s001.zip › cancers-532228-supplementary/Supplementary Figure S1-8/Supplementary Fig S3.pdf]

Supplementary Figure S3

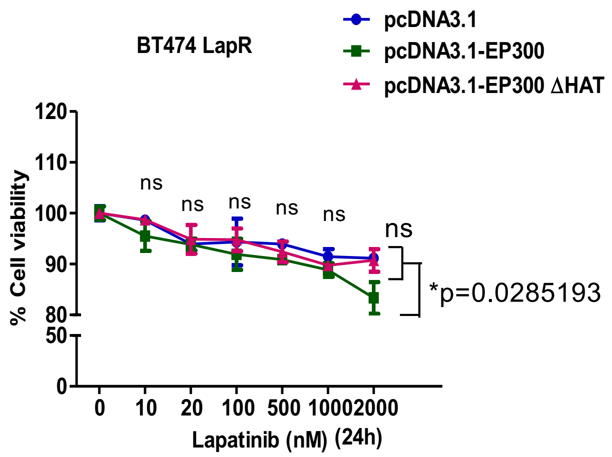

Supplementary figure S3. Overexpression of EP300 or EP300 ΔHAT does not affect Lapatinib resistance in BT474 cells

BT474 Lap^{R} cells transfected with EP300 or EP300 ΔHAT were seeded in 96-well plates and treated with lapatinib at a range of concentration from 10 to 2000 nM. 24 hours after treatment, cells were fixed and stained with the protein-binding dye SRB. Values obtained were normalised against the corresponding untreated controls and presented as percentages. Bars represent the mean ± SEM of three independent transfection experiments (n=3, R=3) and statistical analysis was performed using 1-way ANOVA and tukey post-hoc analysis (ns ,not significant; *p<0.05, significant), showing that EP300 overexpression has an effect on lapatinib sensitivity at 2000 nM.
