# Supplementary material for: EP300 and SIRT1/6 Co-Regulate Lapatinib Sensitivity Via Modulating FOXO3-Acetylation and Activity in Breast Cancer
Source: Cancers (Basel). 2019 Jul 28;11(8):1067. doi: 10.3390/cancers11081067 (PMC6721388; doi:10.3390/cancers11081067)
Supplement: Supplementary file 1 [file cancers-11-01067-s001.zip › cancers-532228-supplementary/Supplementary Figure S1-8/Supplementary Fig S4.pdf]

Supplementary Fig. S4A

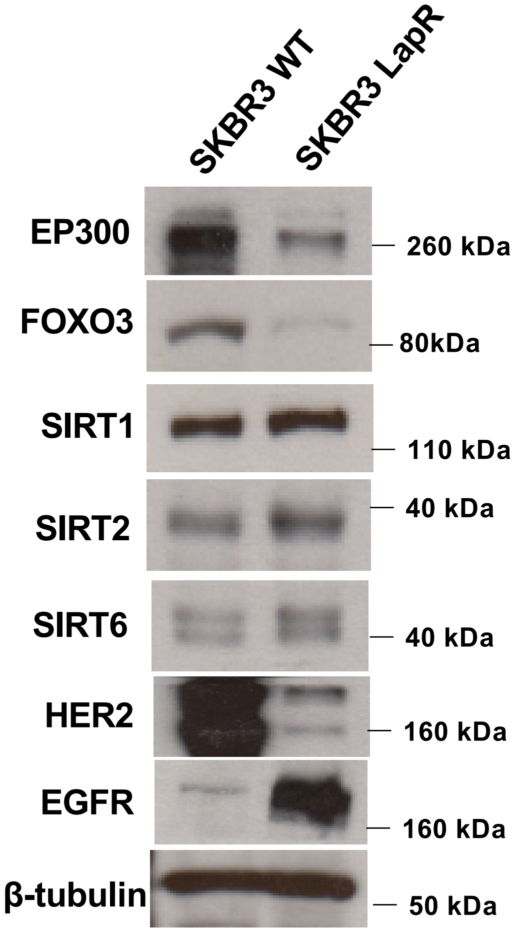

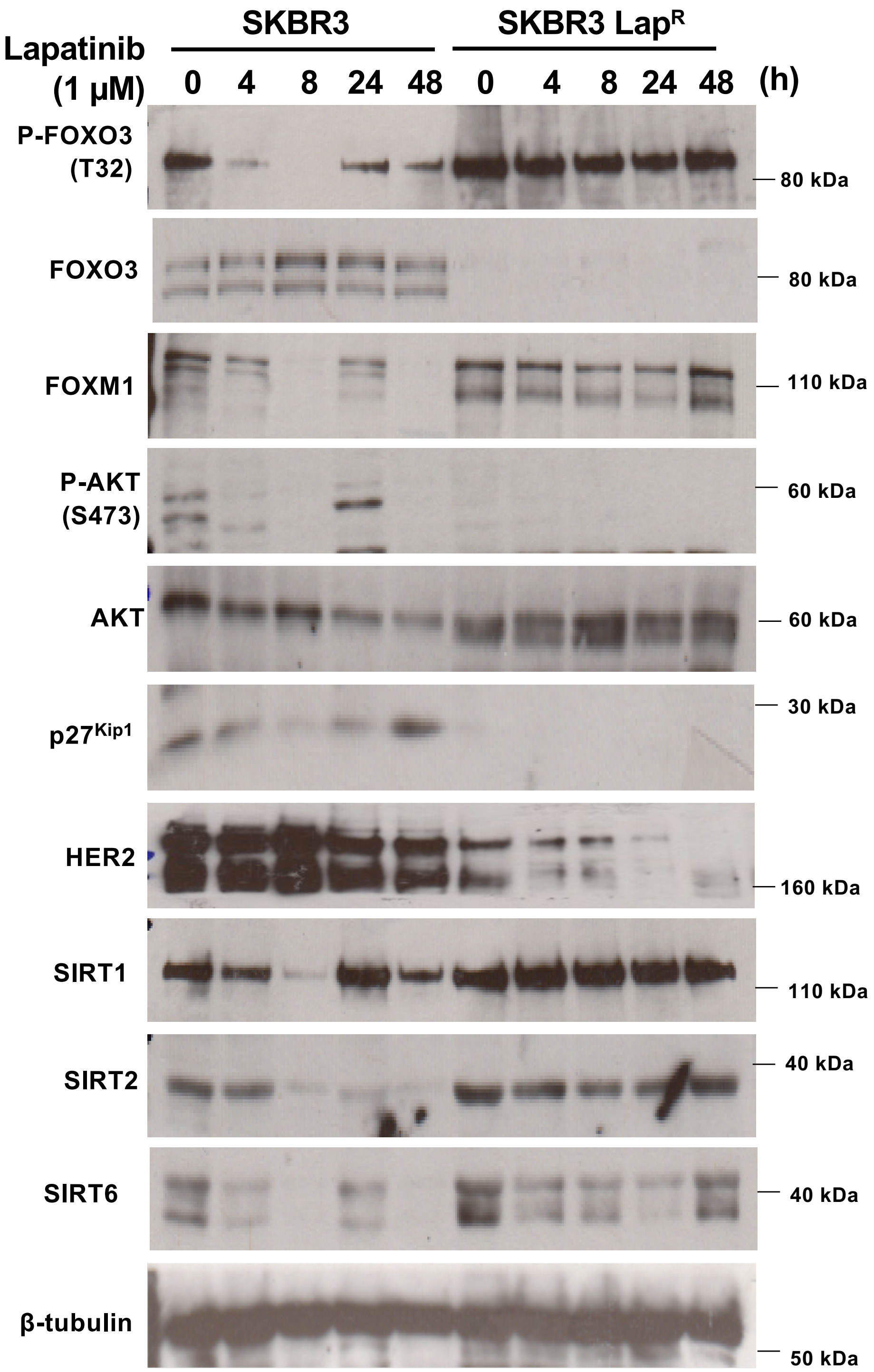

Supplementary Fig. S4A

The sensitive (SKBR3) and lapatinib resistant SKBR3 Lap^{R} cells were either left untreated or treated with 1 µM Lapatinib for the mentioned time points. Protein lysates from whole-cell extracts were then analysed by western blotting using the antibodies against the proteins indicated. Molecular weight markers are shown.

Supplementary Fig. S4B

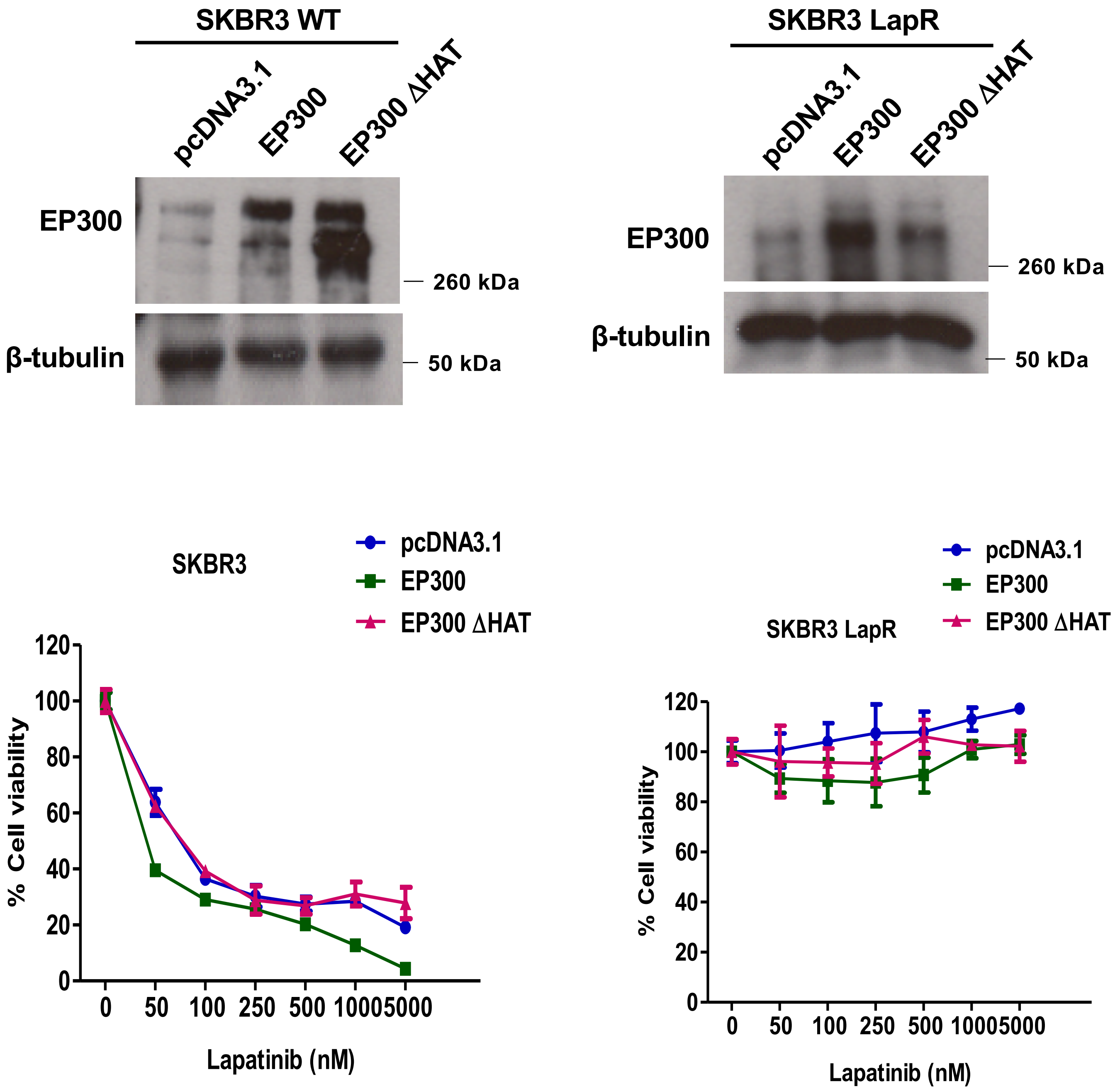

Supplementary Fig. S4B

SKBR3 and SKBR3 Lap^R cells were transiently transfected with the empty vector pcDNA3.1, the plasmid encoding wild-type EP300, or the one encoding for its acetyl-transferase mutant derivative (EP300 △HAT). Transiently transfected SKBR3 WT and LapR cells were seeded in 96-well plates and treated with lapatinib at a range of concentrations from 50 to 5000 nM. Twenty-four hours after treatment, cells were fixed and stained with the protein-binding dye SRB. Values obtained were normalized against the corresponding untreated controls and presented as percentages. Data represent means ± SEM (2-way ANOVA; non-significant, ns; significant, **P<0.01). Moreover, proteins were obtained from whole cell extracts after 24 hours following transfection. Western blotting was performed using the protein lysates to assess the expression levels of EP300.
