# Supplementary material for: EP300 and SIRT1/6 Co-Regulate Lapatinib Sensitivity Via Modulating FOXO3-Acetylation and Activity in Breast Cancer
Source: Cancers (Basel). 2019 Jul 28;11(8):1067. doi: 10.3390/cancers11081067 (PMC6721388; doi:10.3390/cancers11081067)
Supplement: Supplementary file 1 [file cancers-11-01067-s001.zip › cancers-532228-supplementary/Supplementary Figure S1-8/Supplementary Fig S5.pdf]

Supplementary Figure S5

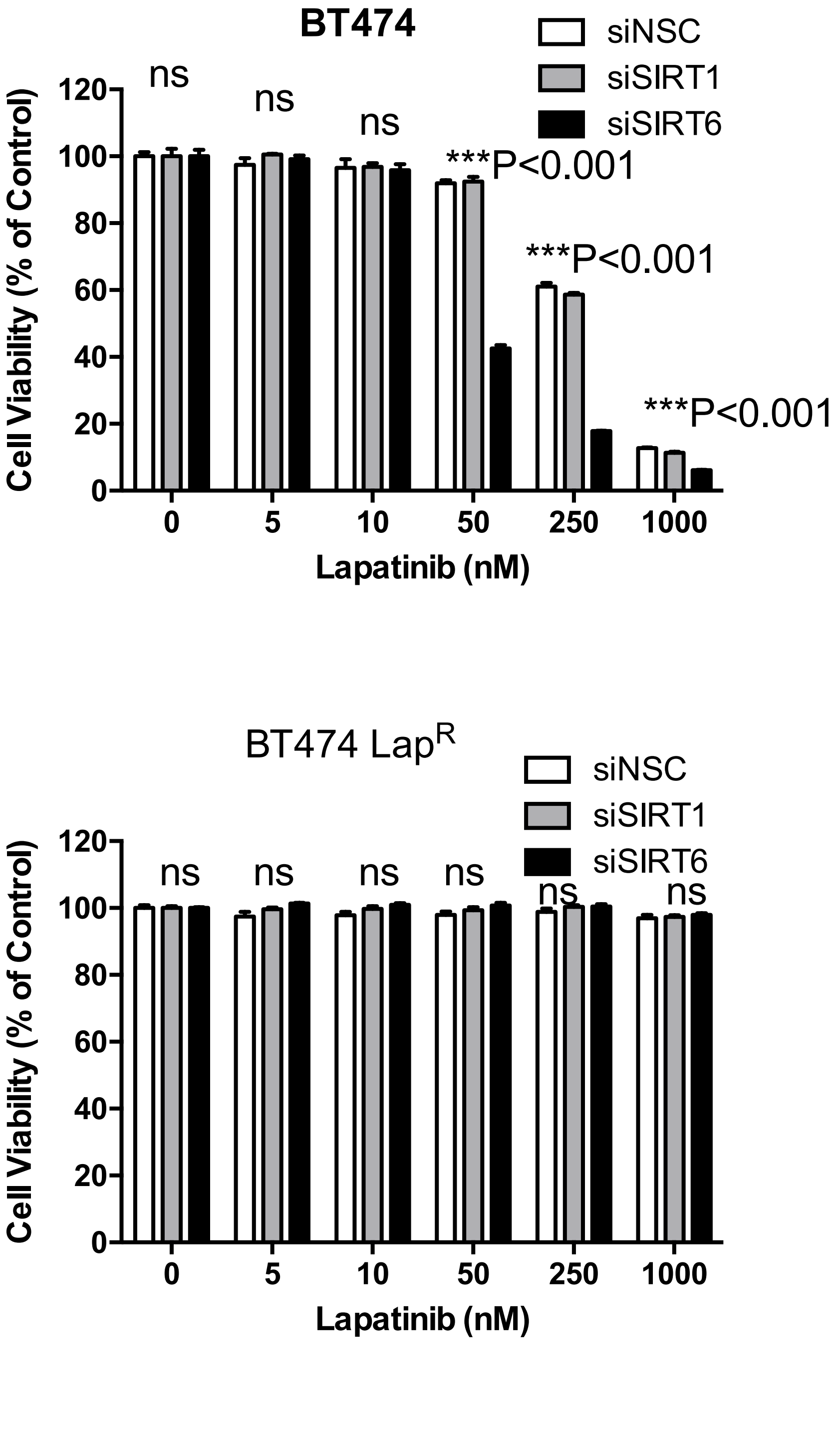

Supplementary figure S5. Silencing of SIRT6 increases lapatinib cytotoxicity in BT474 cells. Transiently transfected BT474 and BT474 Lap^R cells were seeded in 96-well plates and treated with lapatinib at a range of concentrations from 10 to 2000 nM. Twenty-four hours after treatment, cells were fixed and stained with the protein-binding dye SRB. Values obtained were normalized against the corresponding untreated controls (100%) and presented as percentages. Data represent means ± SEM (1-way ANOVA; non-significant, ns; significant, ***P<0.001), showing that BT474 becomes lapatinib sensitive at a range of concentrations from 50 to 2000 after SIRT6 depletion.
