# Supplementary material for: EP300 and SIRT1/6 Co-Regulate Lapatinib Sensitivity Via Modulating FOXO3-Acetylation and Activity in Breast Cancer
Source: Cancers (Basel). 2019 Jul 28;11(8):1067. doi: 10.3390/cancers11081067 (PMC6721388; doi:10.3390/cancers11081067)
Supplement: Supplementary file 1 [file cancers-11-01067-s001.zip › cancers-532228-supplementary/Supplementary Figure S1-8/Supplementary Fig S6.pdf]

Supplementary Fig. S6

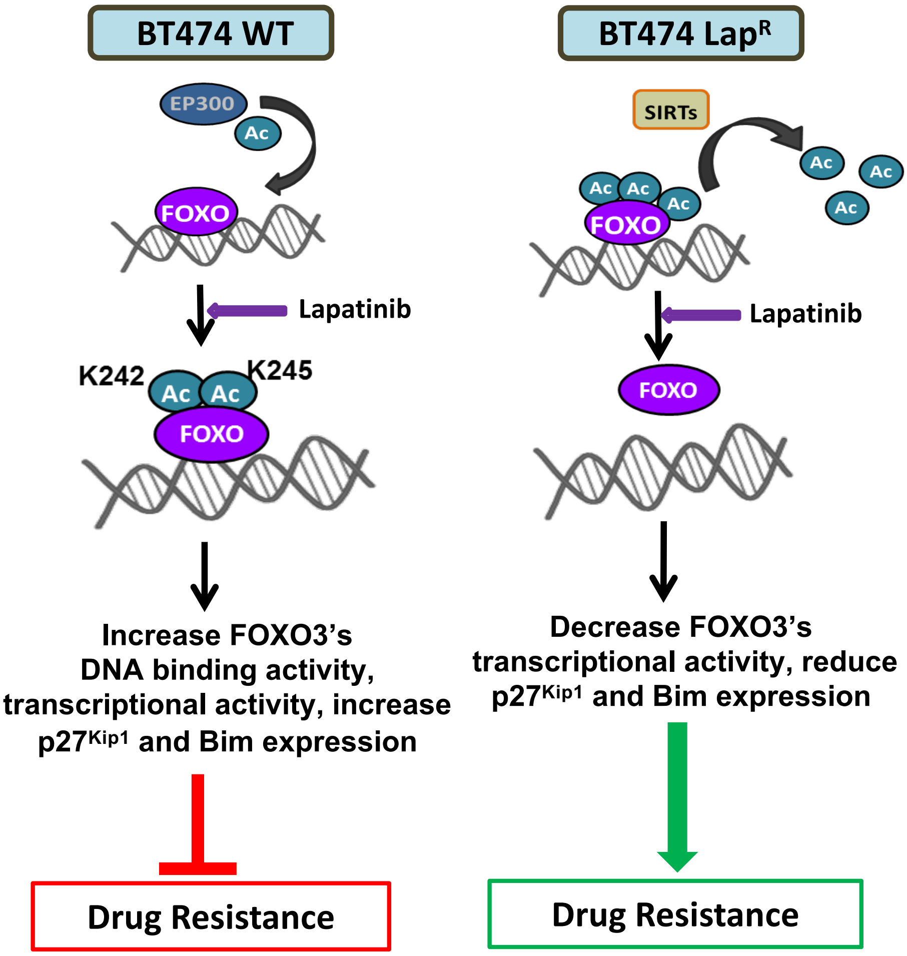

Supplementary figure S6.
Schematic model showing the regulation of FOXO3 acetylation by EP300 and SIRT1/6 and its subsequent outcome on Lapatinib sensitivity and resistance in breast cancer cells
