# Supplementary material for: EP300 and SIRT1/6 Co-Regulate Lapatinib Sensitivity Via Modulating FOXO3-Acetylation and Activity in Breast Cancer
Source: Cancers (Basel). 2019 Jul 28;11(8):1067. doi: 10.3390/cancers11081067 (PMC6721388; doi:10.3390/cancers11081067)
Supplement: Supplementary file 1 [file cancers-11-01067-s001.zip › cancers-532228-supplementary/Supplementary Figure S1-8/Supplementary Fig S7.pdf]

Supplementary Fig. S7A

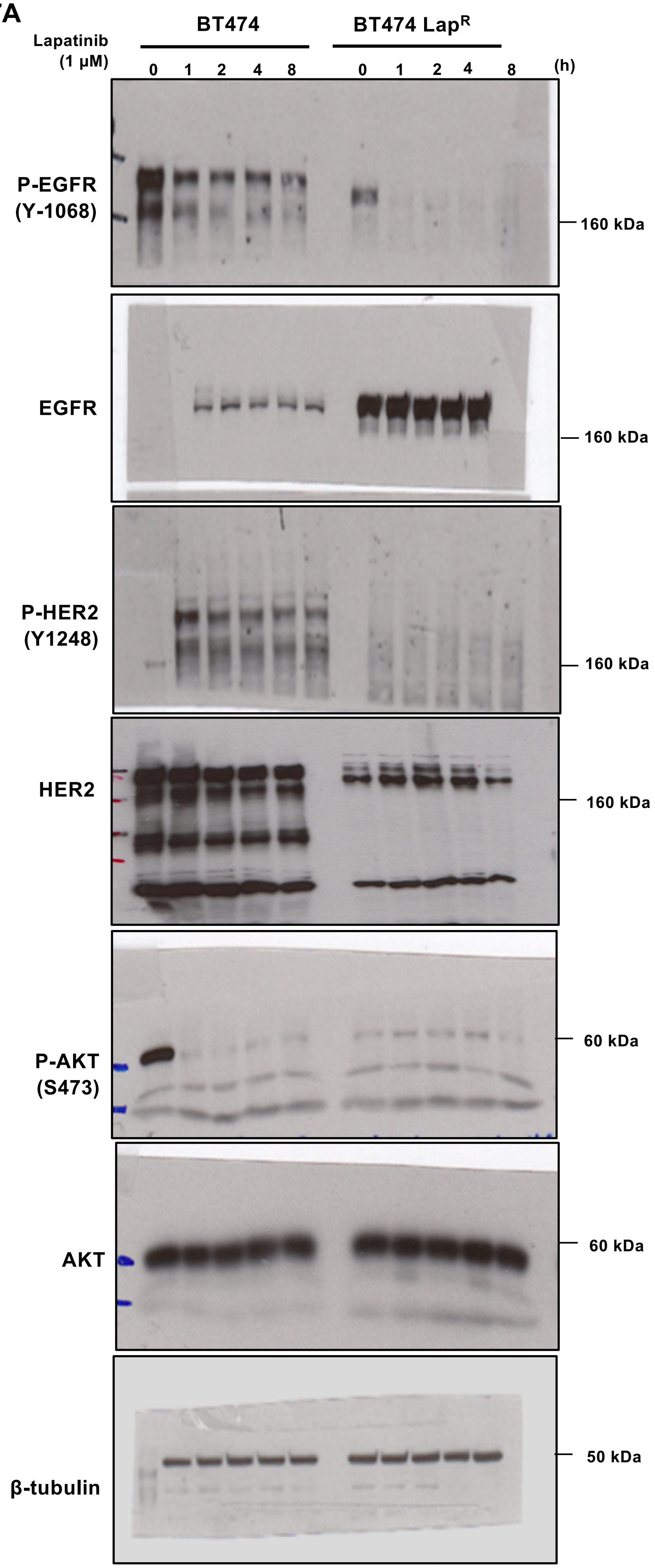

Supplementary Figure S7A

Original blots for the cropped/composite Fig. 1A

Supplementary Fig. S7B

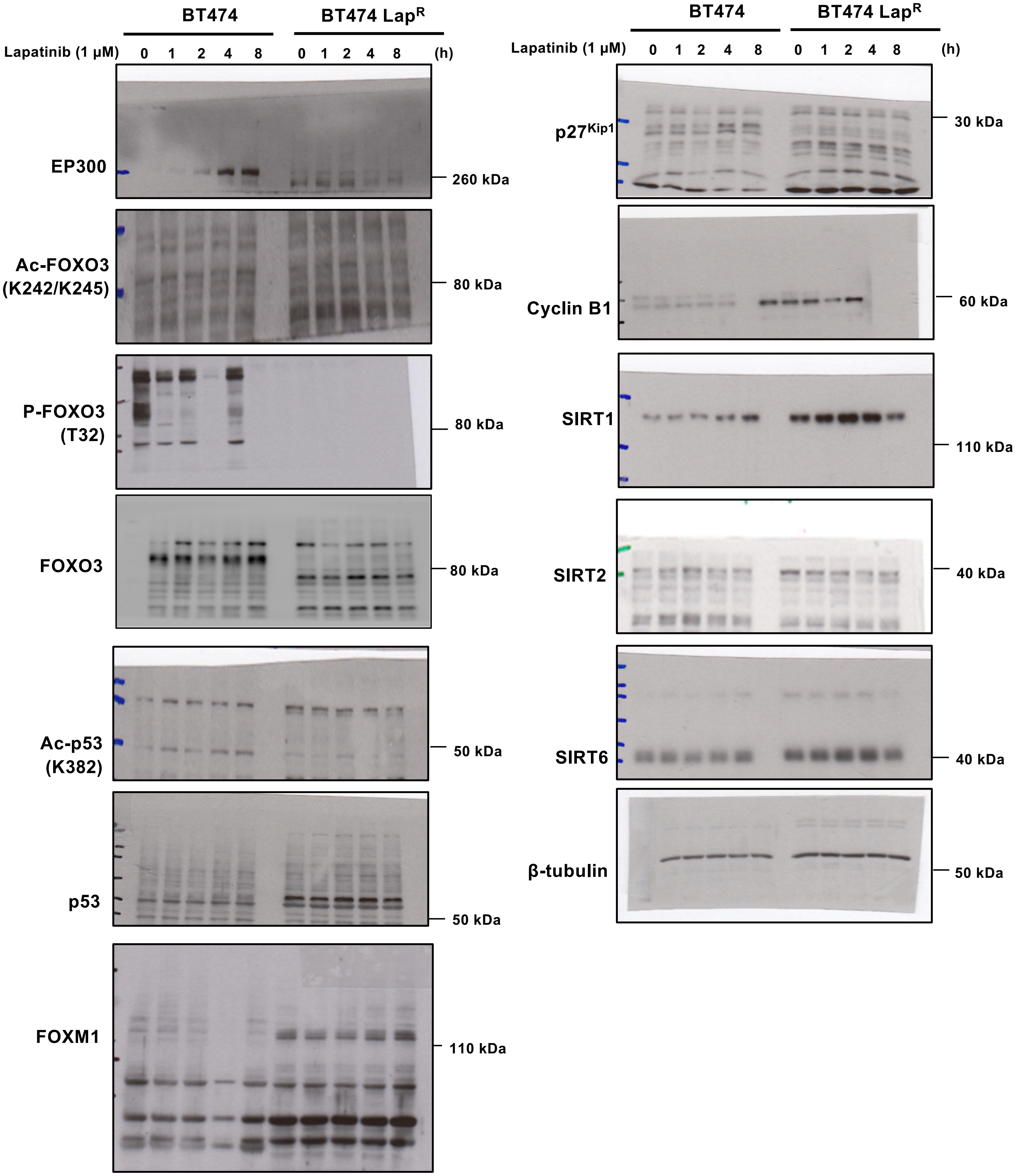

Supplementary Figure S7B

Original blots for the cropped/composite Fig. 1B

Supplementary Fig. S7C

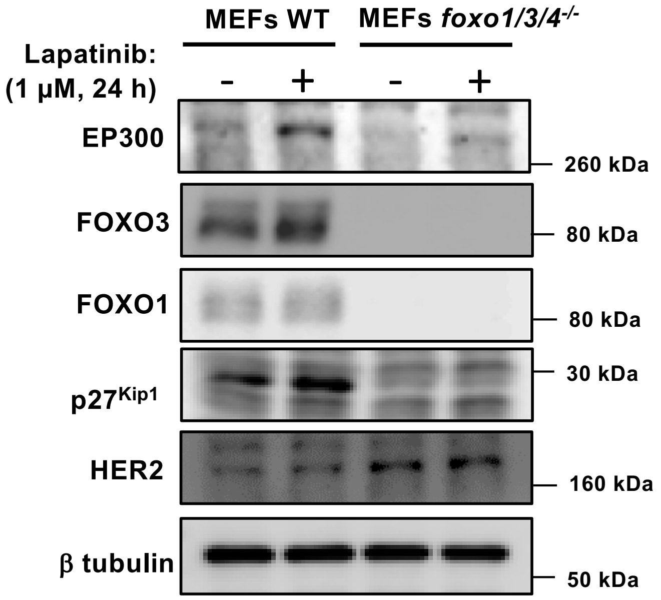

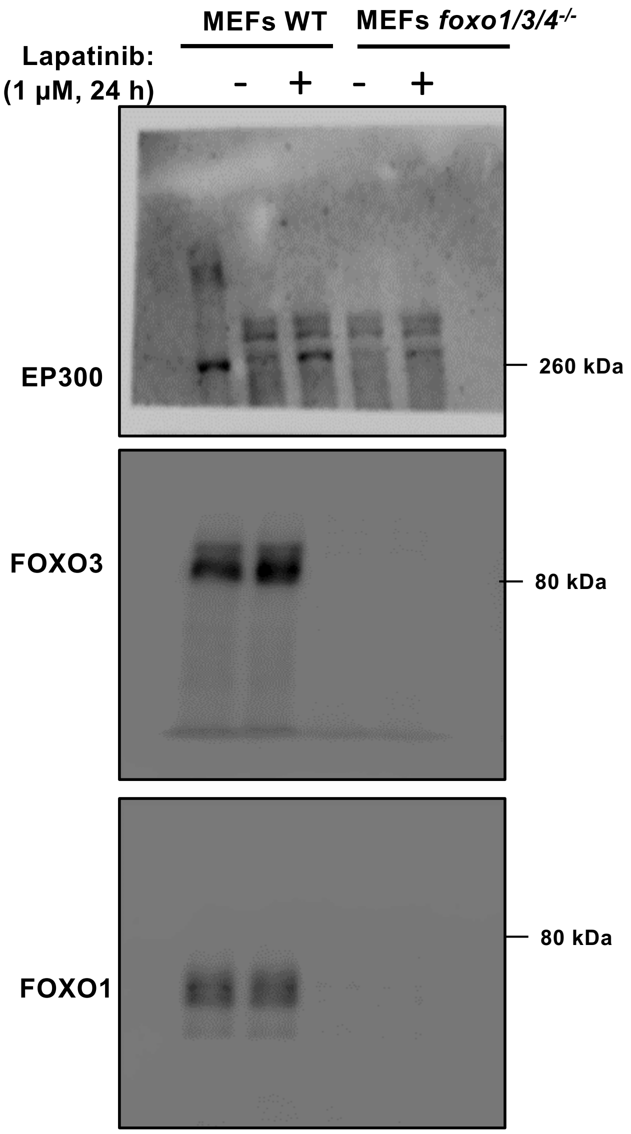

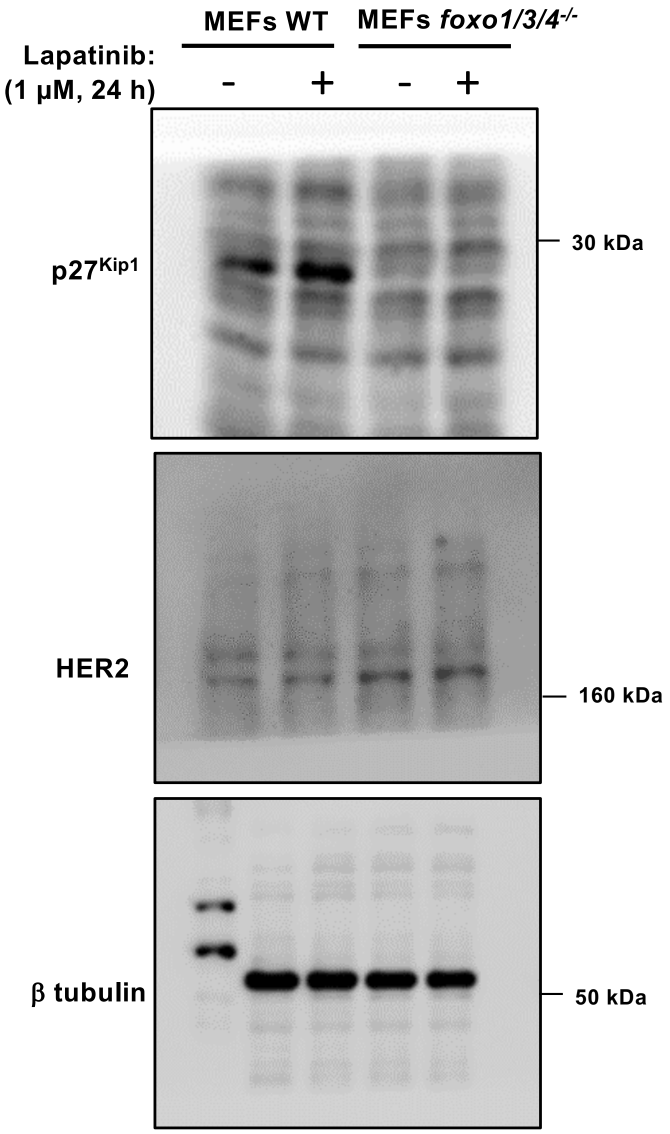

Supplementary Figure S7C

Original blots for the cropped/composite Fig. 2B

Supplementary Fig. S7D

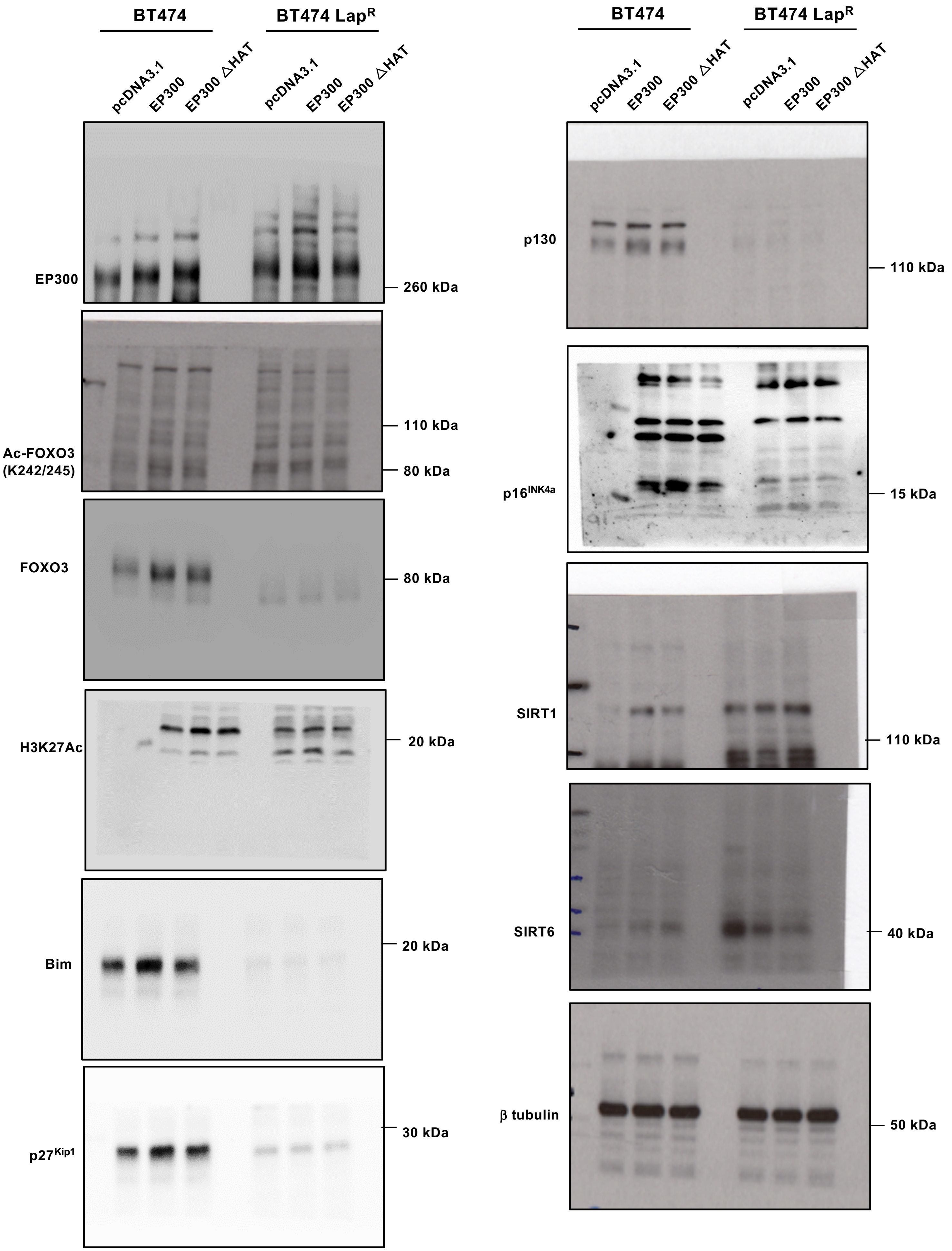

Supplementary Figure S7D

Original blots for the cropped/composite Fig. 4A

Supplementary Fig. S7E

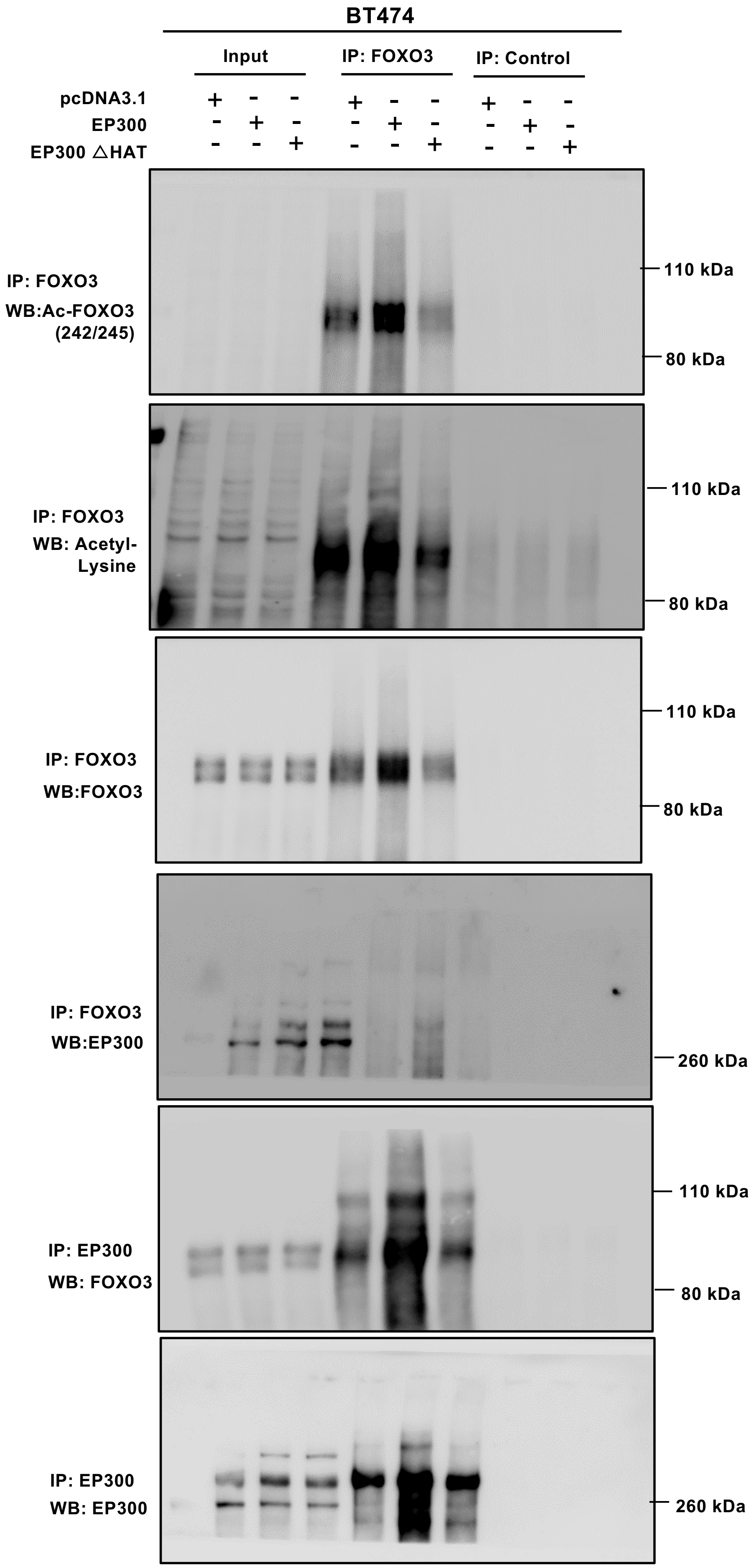

Supplementary Figure S7E

Original blots for the cropped/composite Fig. 4B

Supplementary Fig. S7F

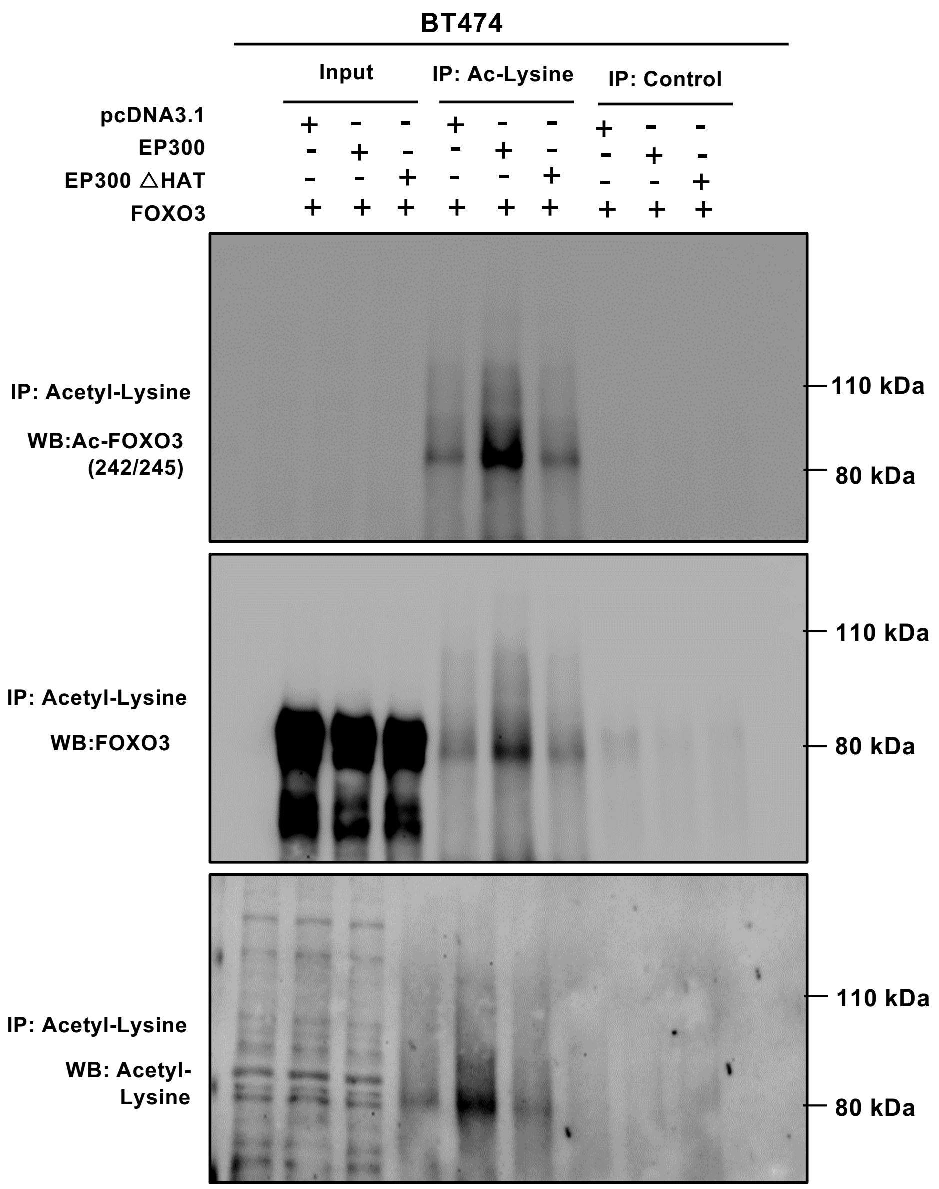

Supplementary Figure S7F
Original blots for the cropped/composite Fig. 4C

Supplementary Fig. S7G

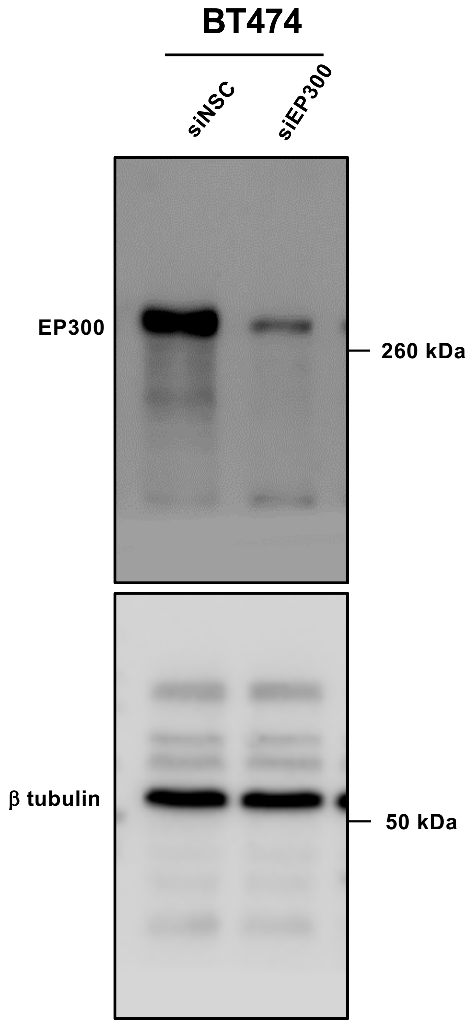

Supplementary Figure S7G
Original blots for the cropped/composite Fig. 5B

Supplementary Fig. S7H

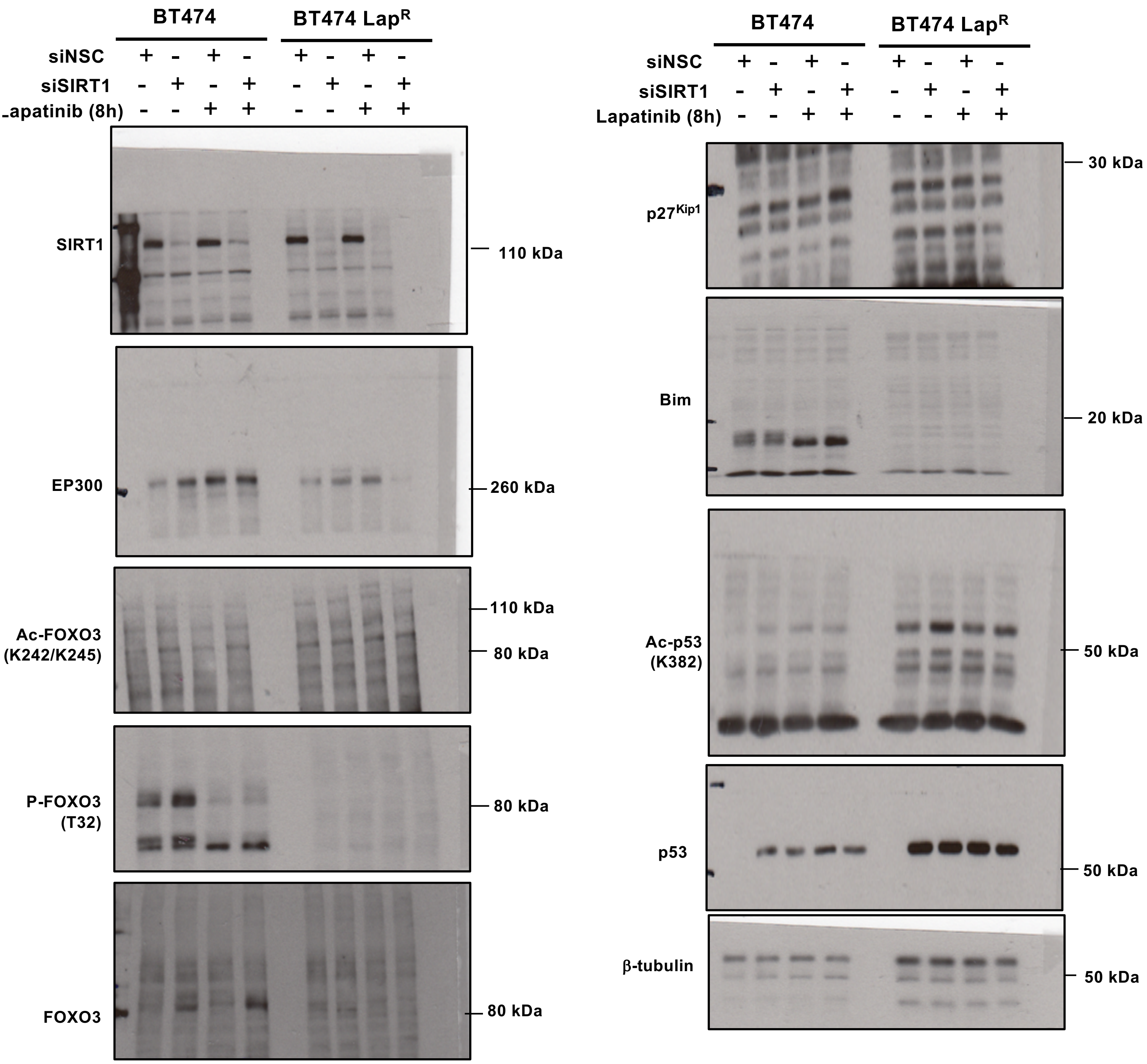

Supplementary Figure S7H
Original blots for the cropped/composite Fig. 6A

Supplementary Fig. S7I

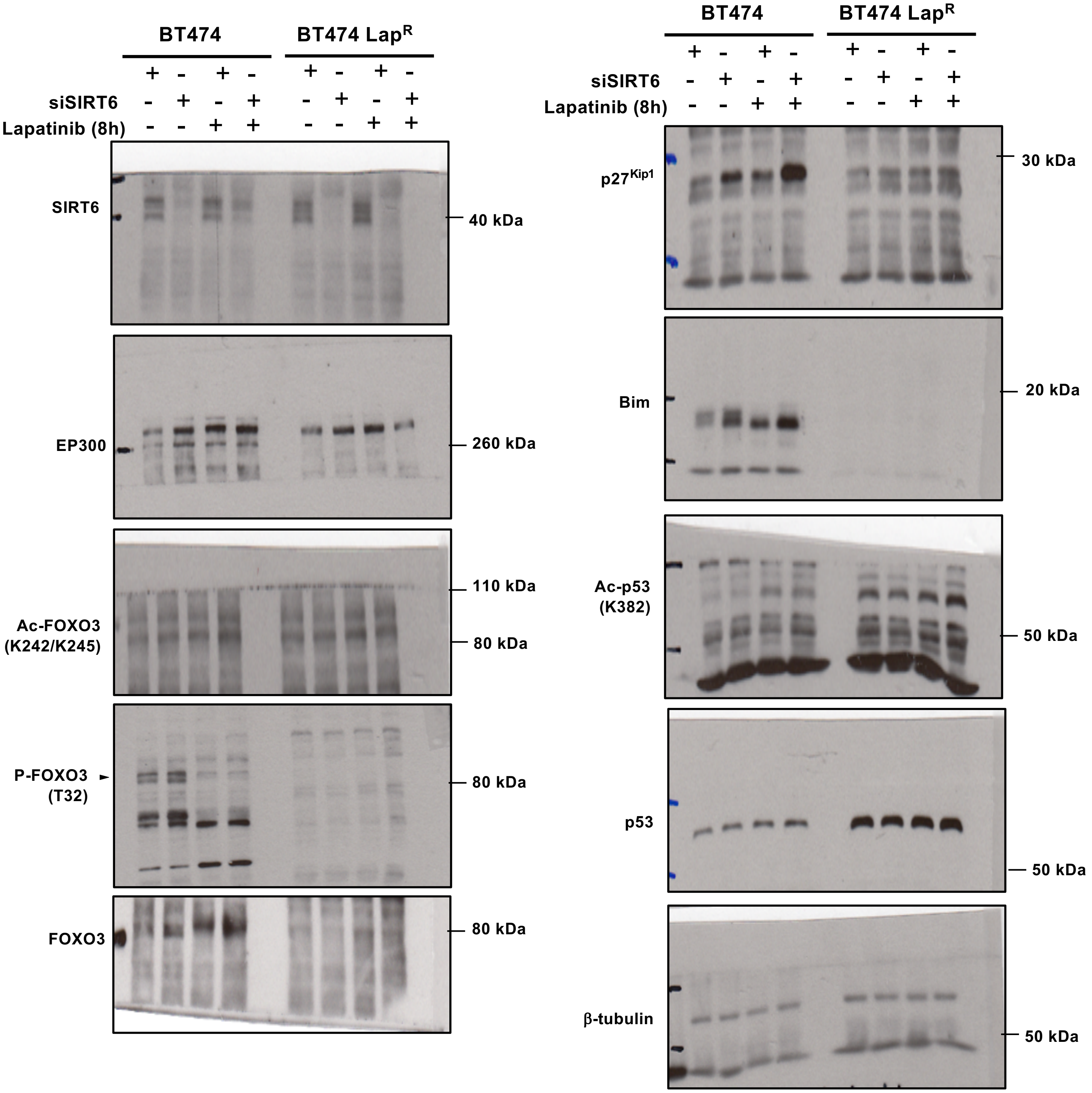

Supplementary Figure S7I
Original blots for the cropped/composite Fig. 6B

Supplementary Fig. S7J

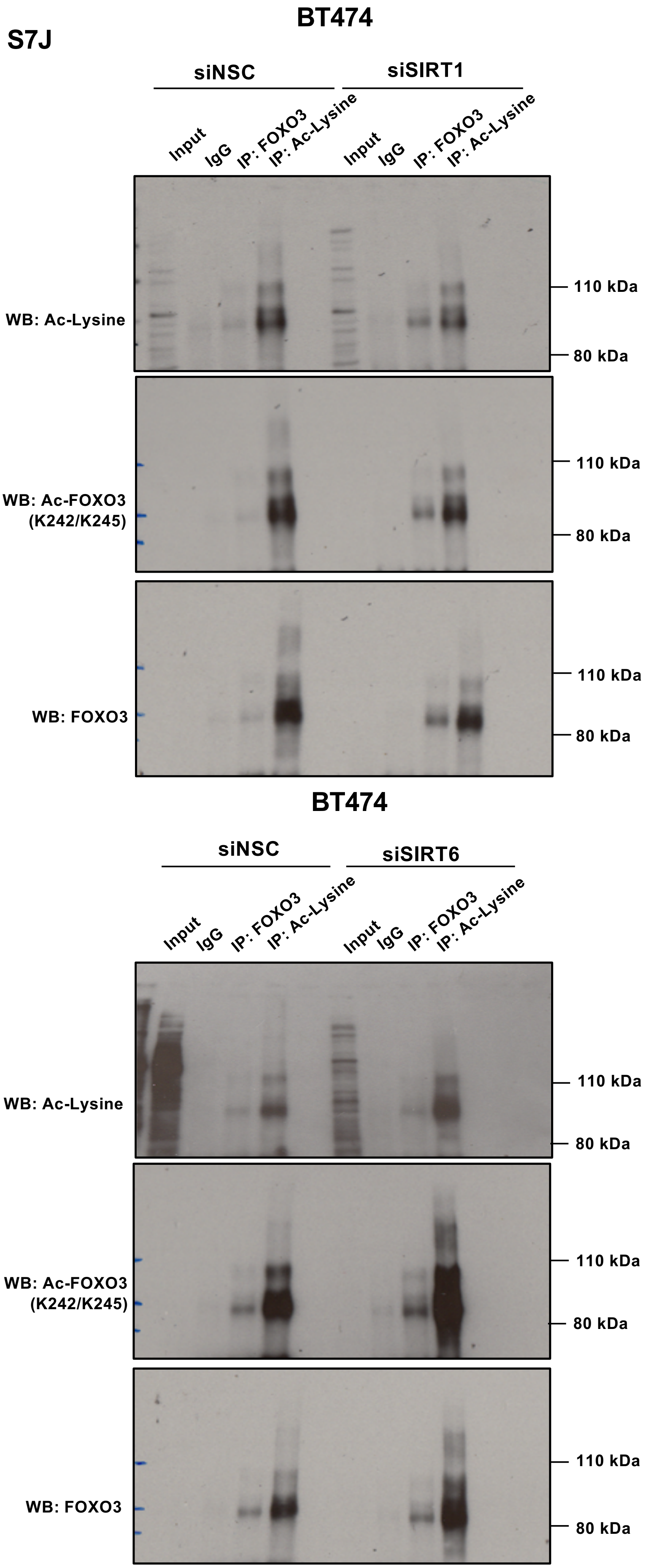

Supplementary Figure S7J

Original blots for the cropped/composite Fig. 6C

Supplementary Fig. S7K

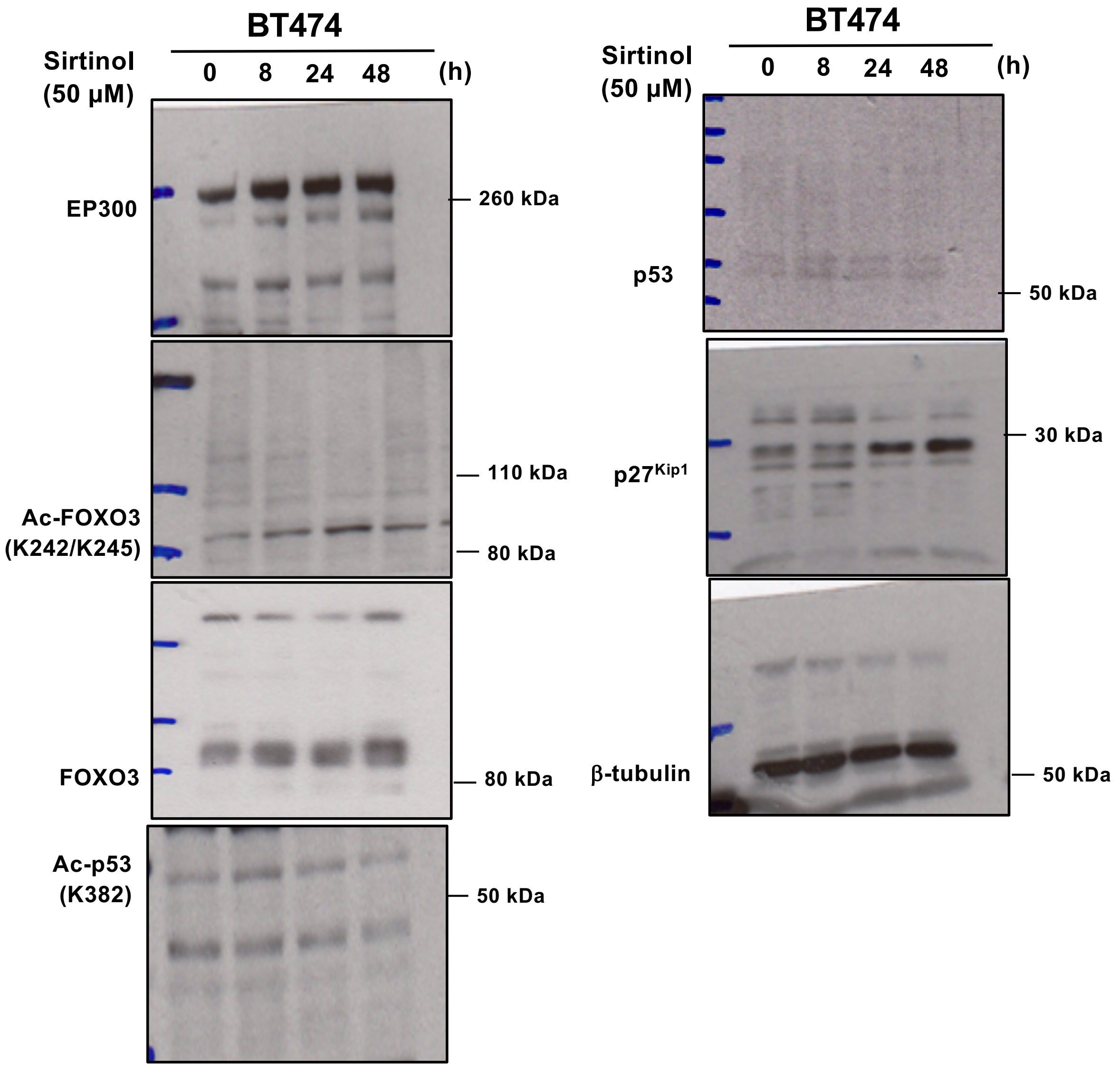

Supplementary Figure S7K

Original blots for the cropped/composite Fig. 7B

Supplementary Fig. S7L

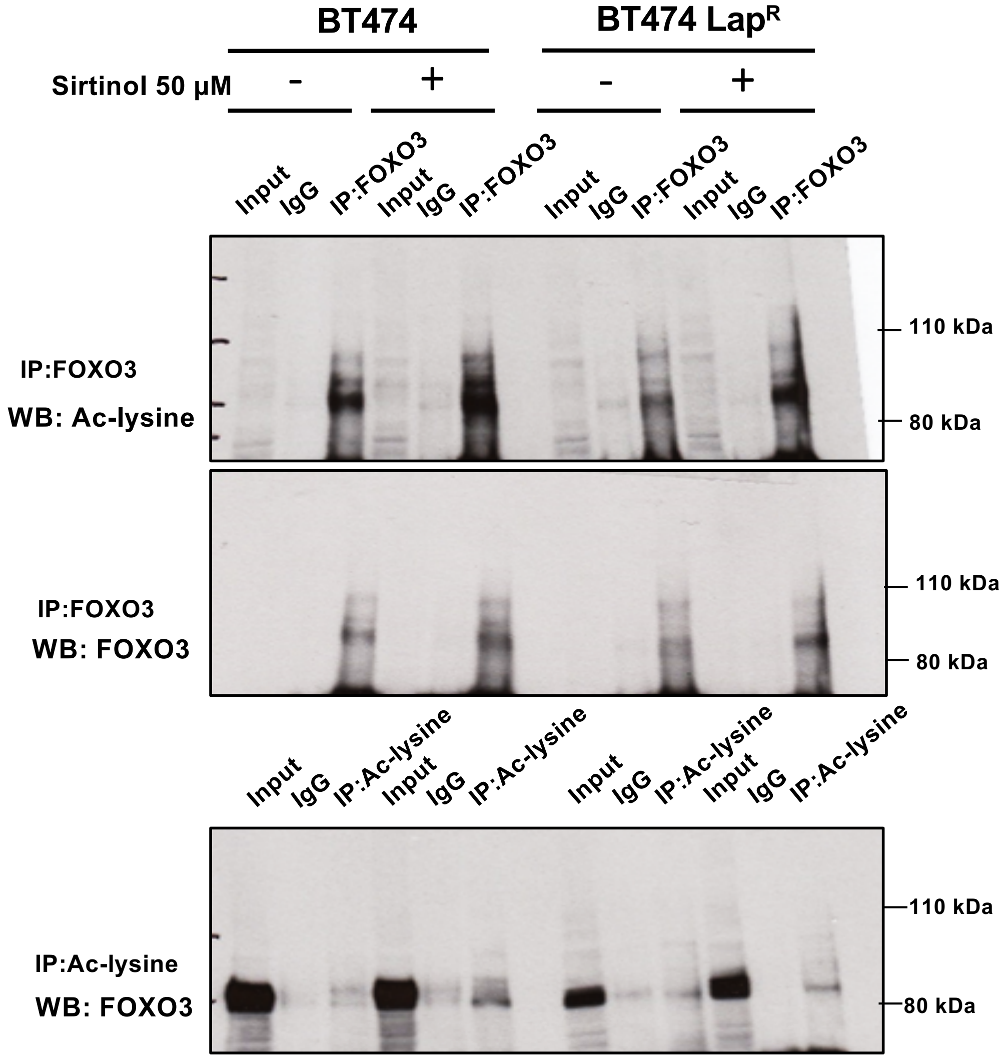

Supplementary Figure S7L

Original blots for the cropped/composite Fig. 7C

Supplementary Fig. S7M

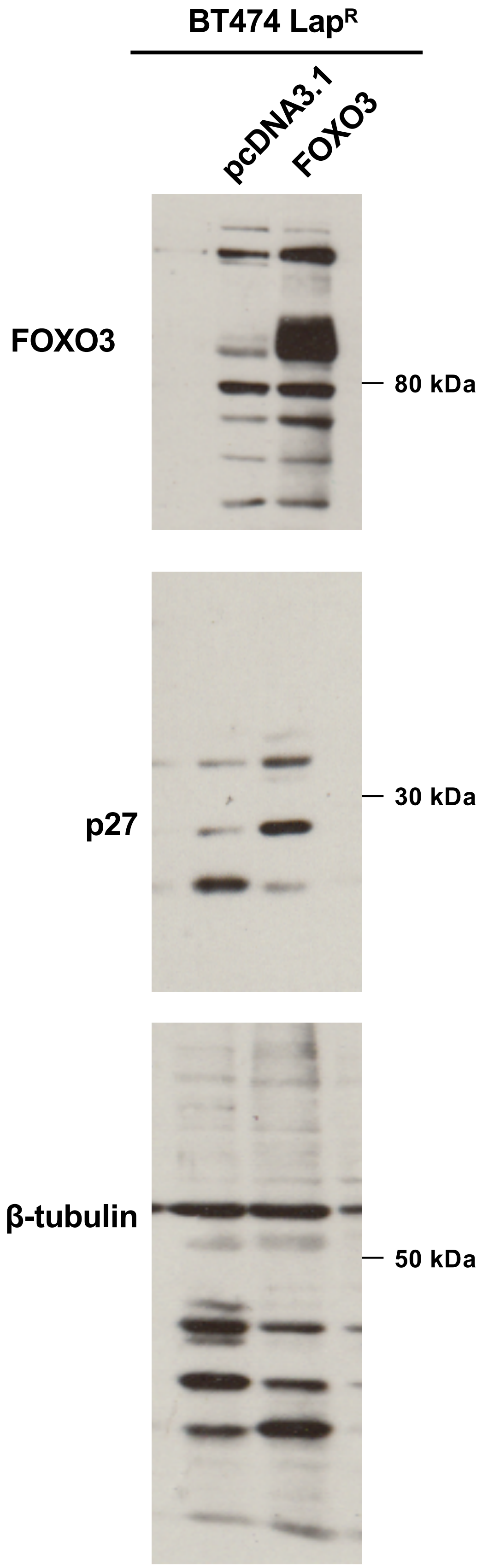

Supplementary Fig. S7M
Original blots for the cropped/composite for Supplementary Fig S2A

Supplementary Fig. S7N

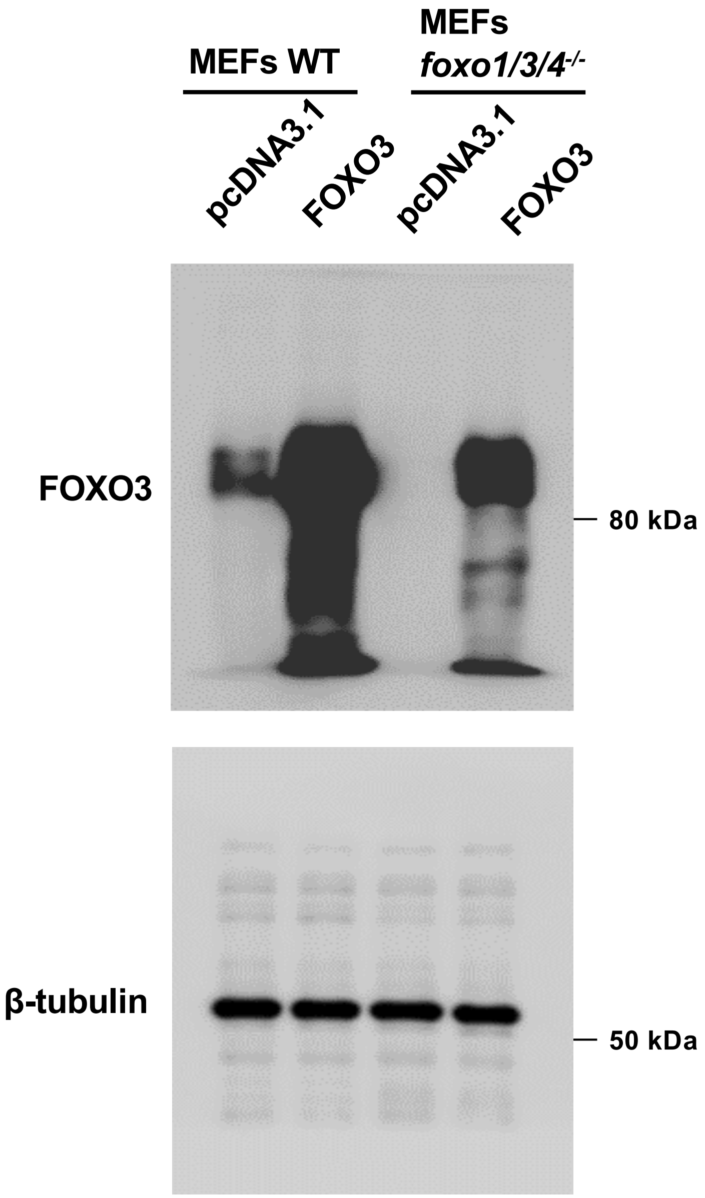

Supplementary Fig. S7N
Original blots for the cropped/composite for Supplementary Fig S2B

Supplementary Fig. S7O

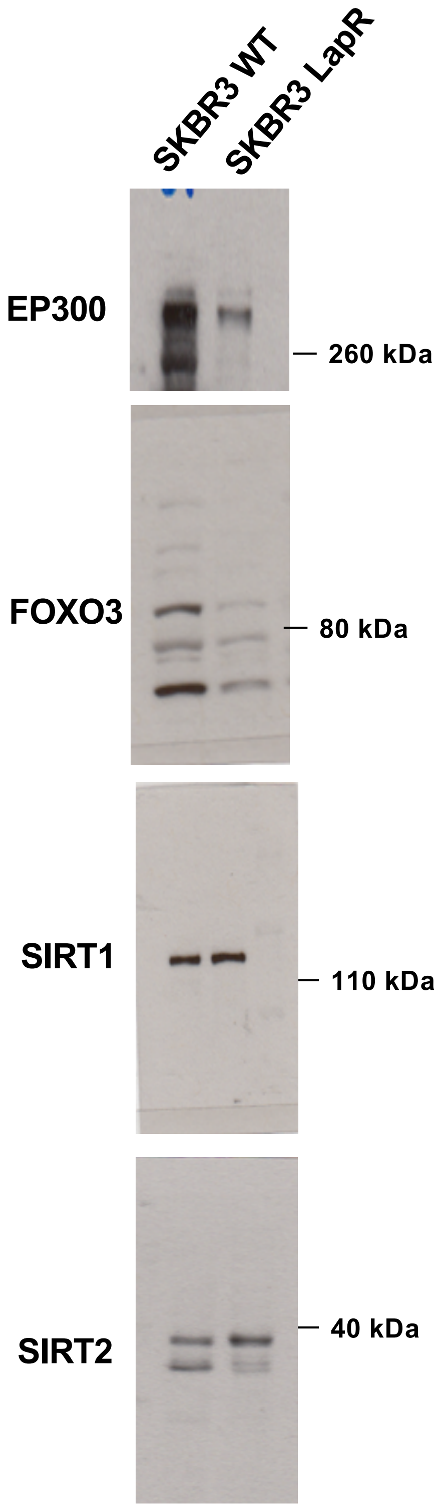

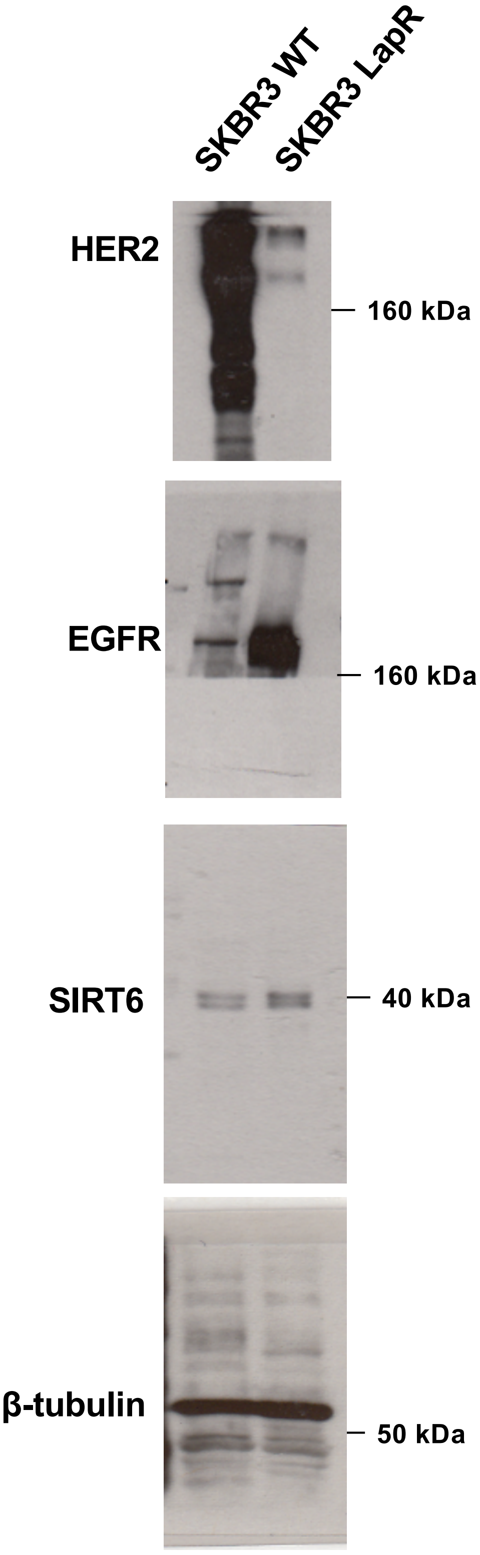

Supplementary Fig. S7O
Original blots for the cropped/composite for Supplementary Fig S4A

Supplementary Fig. S7P

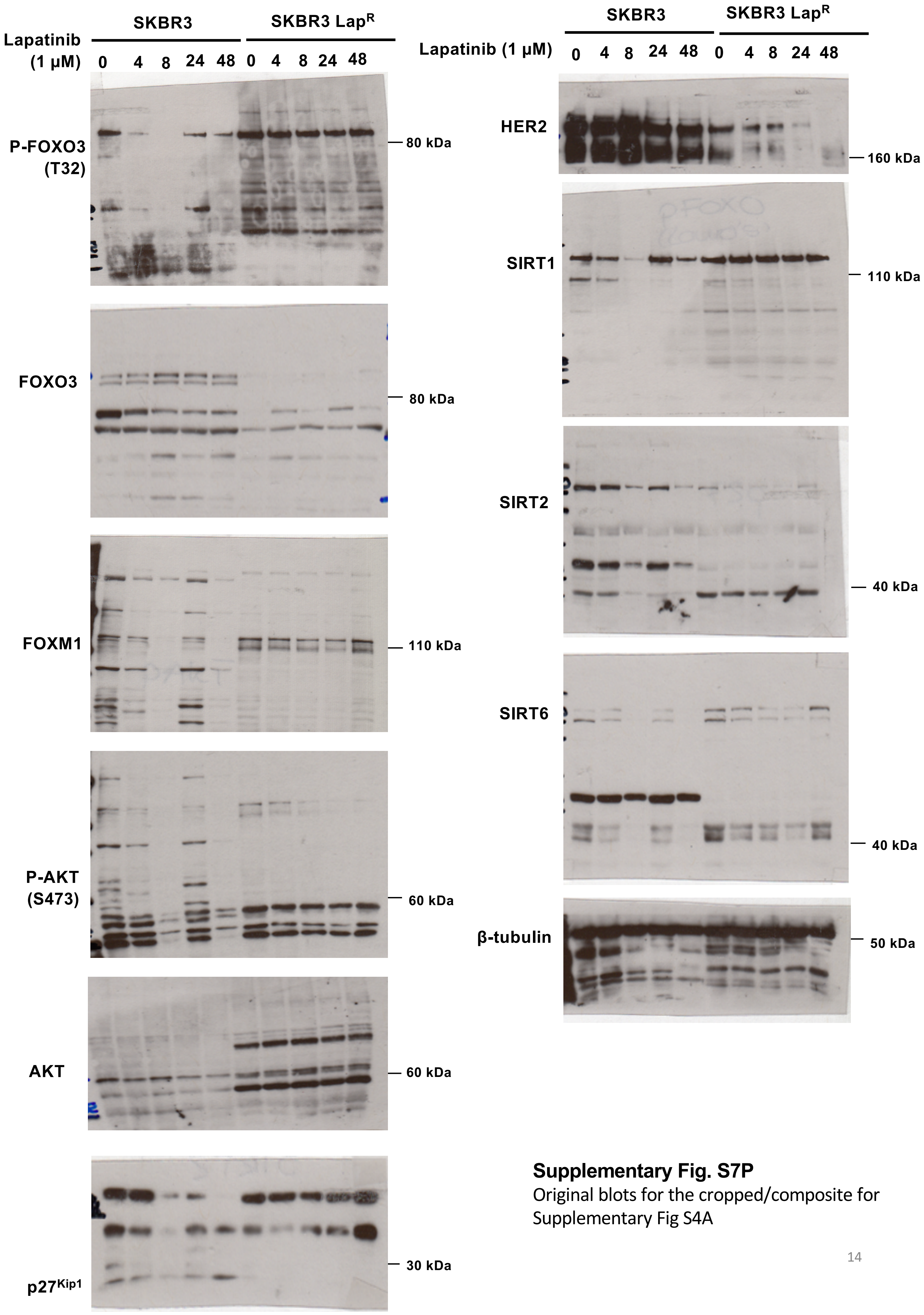

Supplementary Fig. S7P
Original blots for the cropped/composite for Supplementary Fig S4A

Supplementary Fig. S7Q

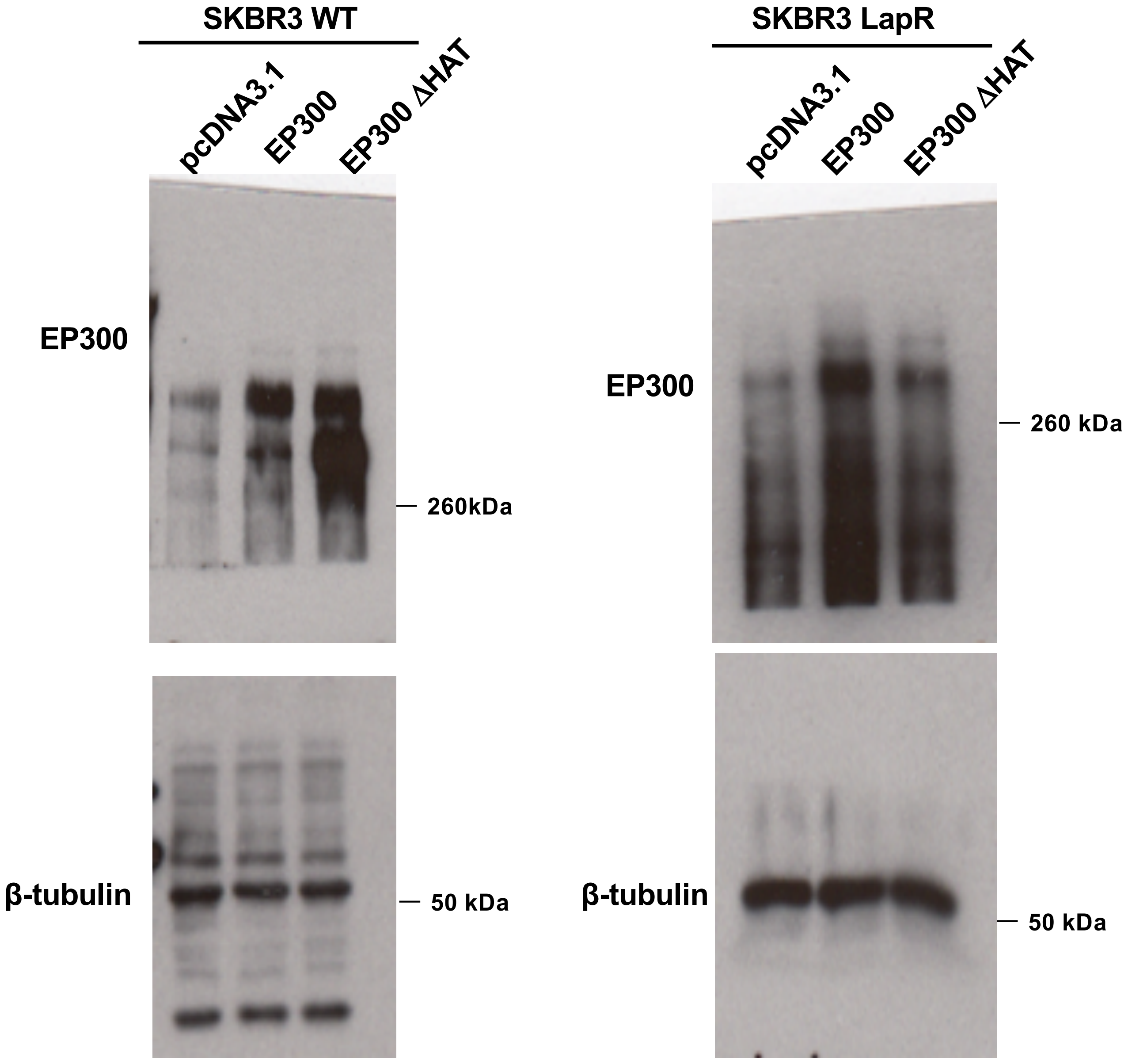

Supplementary Fig. S7Q
Original blots for the cropped/composite for Supplementary Fig S4B
