# Supplementary material for: EP300 and SIRT1/6 Co-Regulate Lapatinib Sensitivity Via Modulating FOXO3-Acetylation and Activity in Breast Cancer
Source: Cancers (Basel). 2019 Jul 28;11(8):1067. doi: 10.3390/cancers11081067 (PMC6721388; doi:10.3390/cancers11081067)
Supplement: Supplementary file 1 [file cancers-11-01067-s001.zip › cancers-532228-supplementary/Supplementary Figure S1-8/Supplementary Fig S8.pdf]

Supplementary Fig. S8A

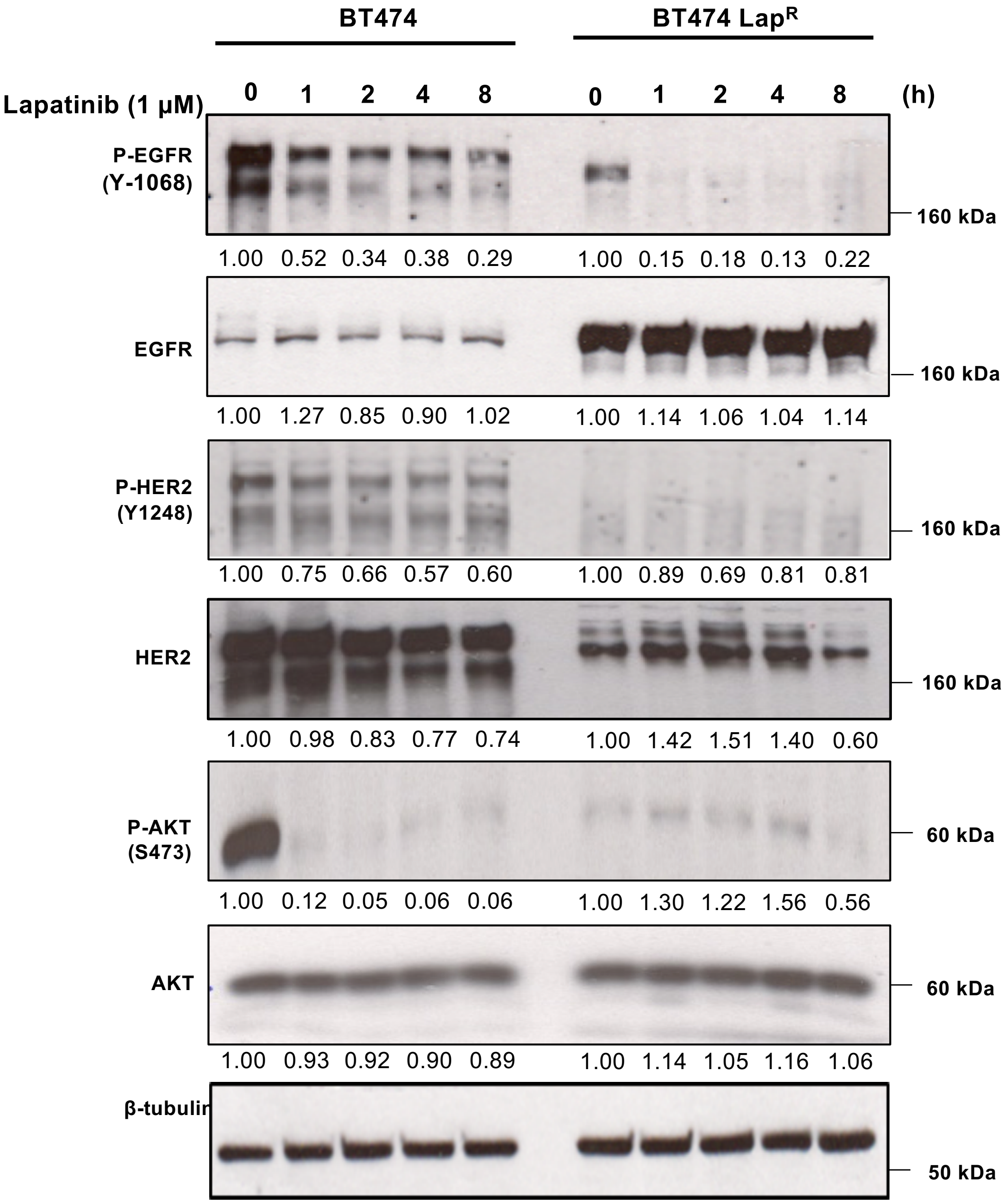

Supplementary Figure S8A.
Protein quantification is performed by ImageJ analysis from Figure 1A

Supplementary Fig. S8B

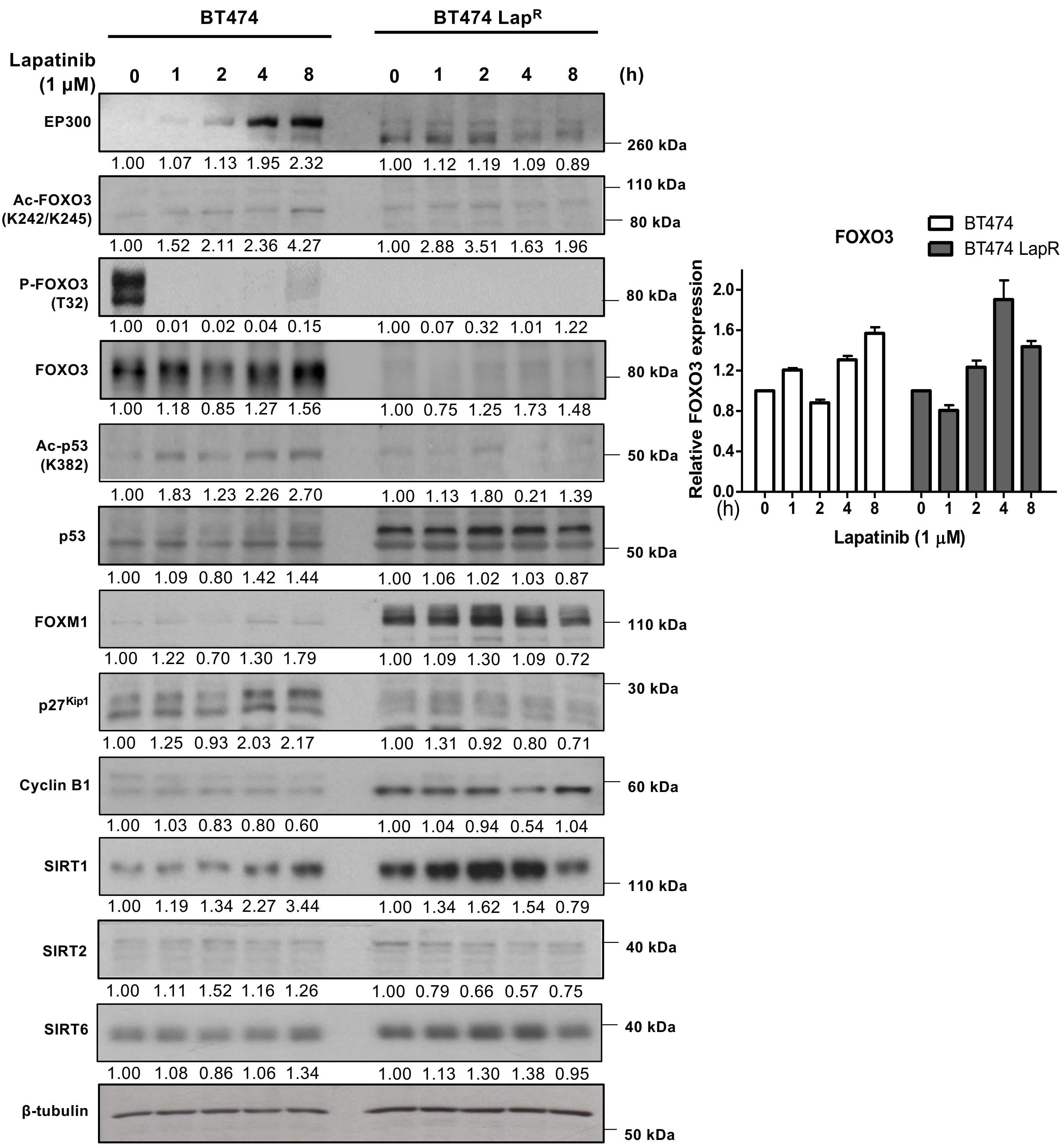

Supplementary Figure S8B.

Protein quantification is performed by ImageJ analysis from Figure 1B (Left panel). FOXO3 protein quantification is shown in bar diagram with mean+/-SEM from three independent experiments (Right Panel).

Supplementary Fig. S8C

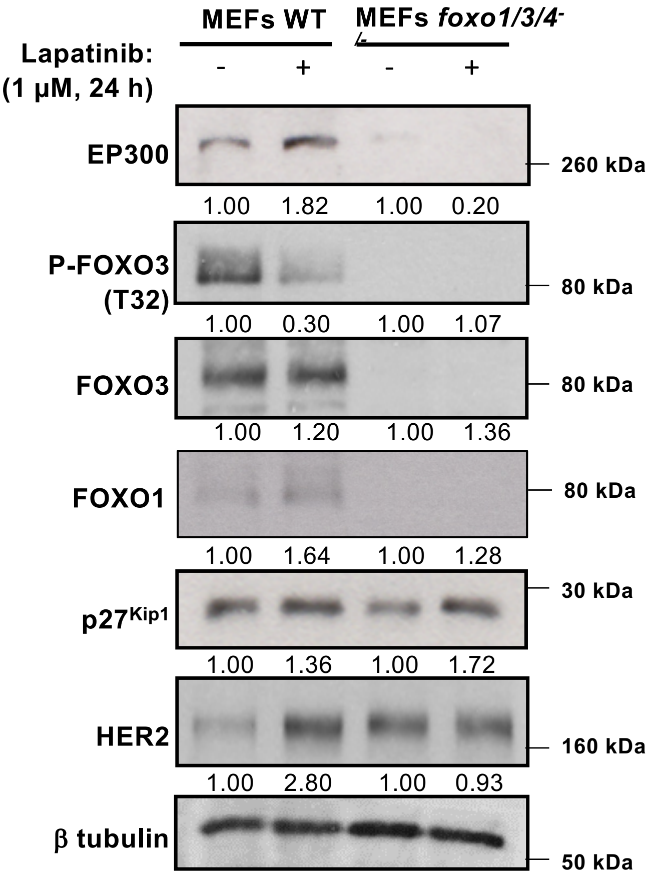

Supplementary Figure S8C
Protein quantification is performed by ImageJ analysis from Figure 2B

Supplementary Fig. S8D

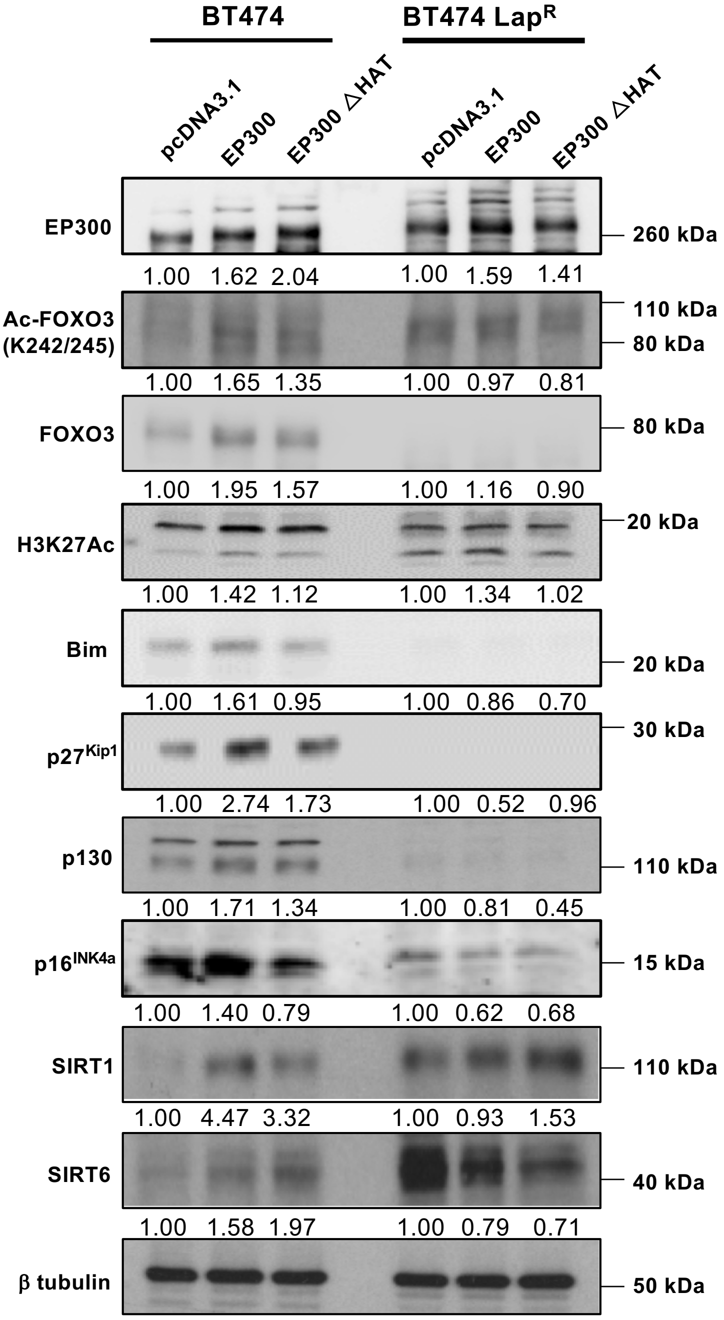

Supplementary Figure S8D

Protein quantification is performed by ImageJ analysis from Figure 4A

Supplementary Fig. S8E

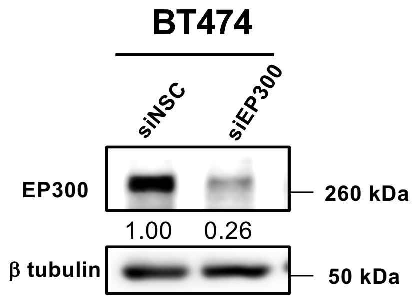

Supplementary Figure S8E

Protein quantification is performed by ImageJ analysis from Figure 5B

Supplementary Fig. S8F

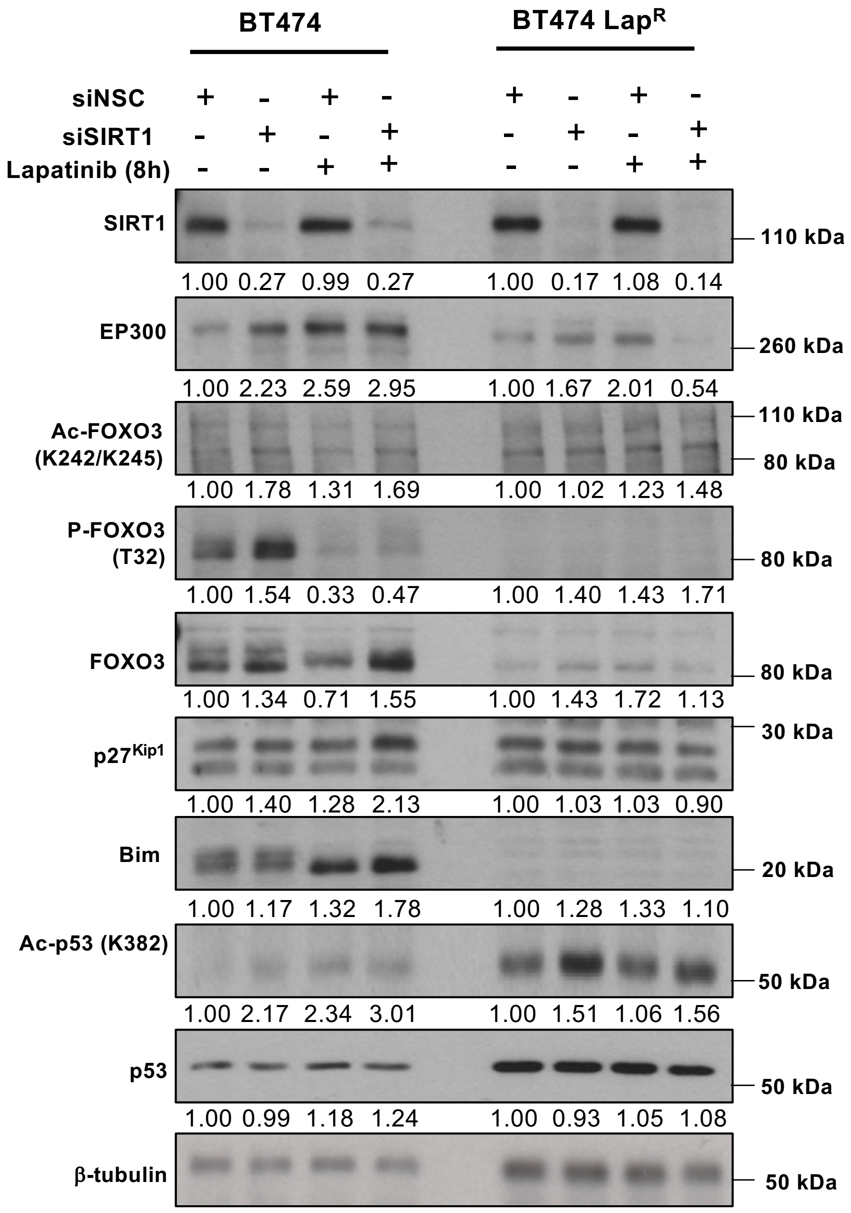

Supplementary Figure S8F

Protein quantification is performed by ImageJ analysis from Figure 6A

Supplementary Fig. S8G

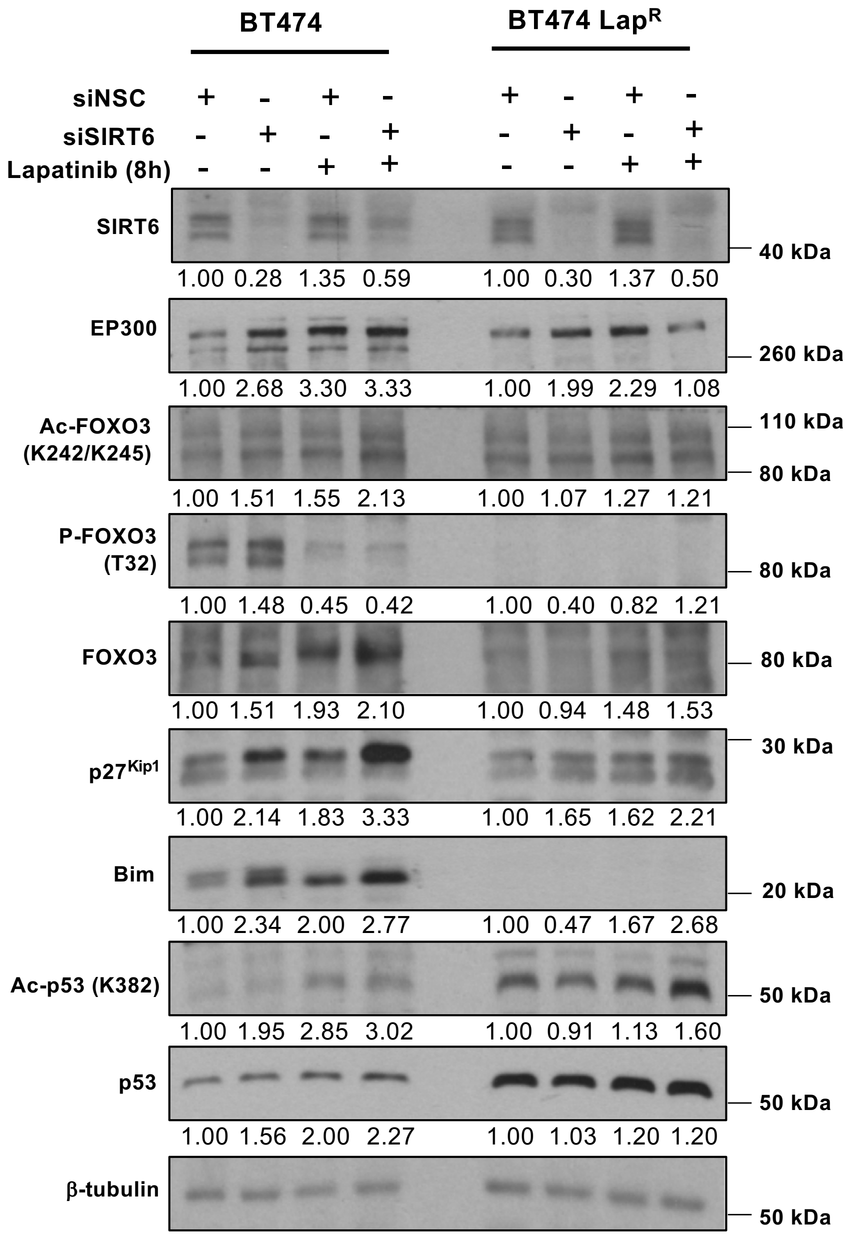

Supplementary Figure S8G

Protein quantification is performed by ImageJ analysis from Figure 6B

Supplementary Fig. S8H

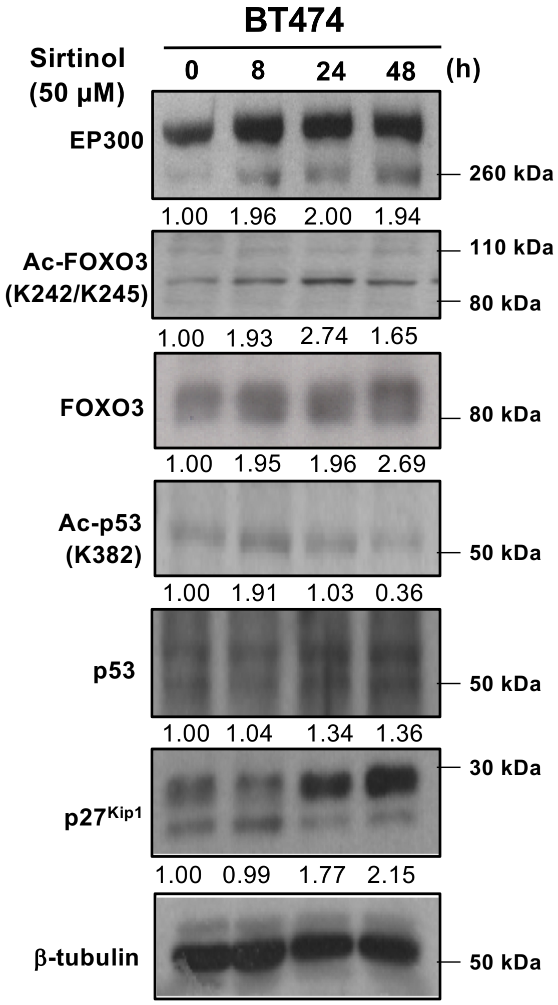

Supplementary Fig. S8I

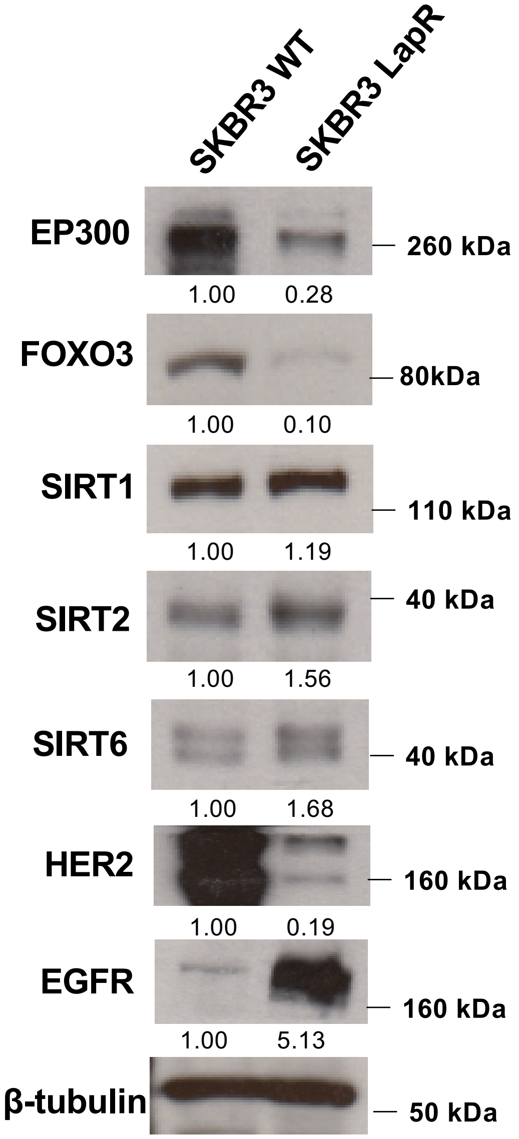

Supplementary Figure S8H
Protein quantification is performed by ImageJ analysis from Figure 7B

Supplementary Figure S8I
Protein quantification is performed by ImageJ analysis from Figure S7A

Supplementary Fig. S8J

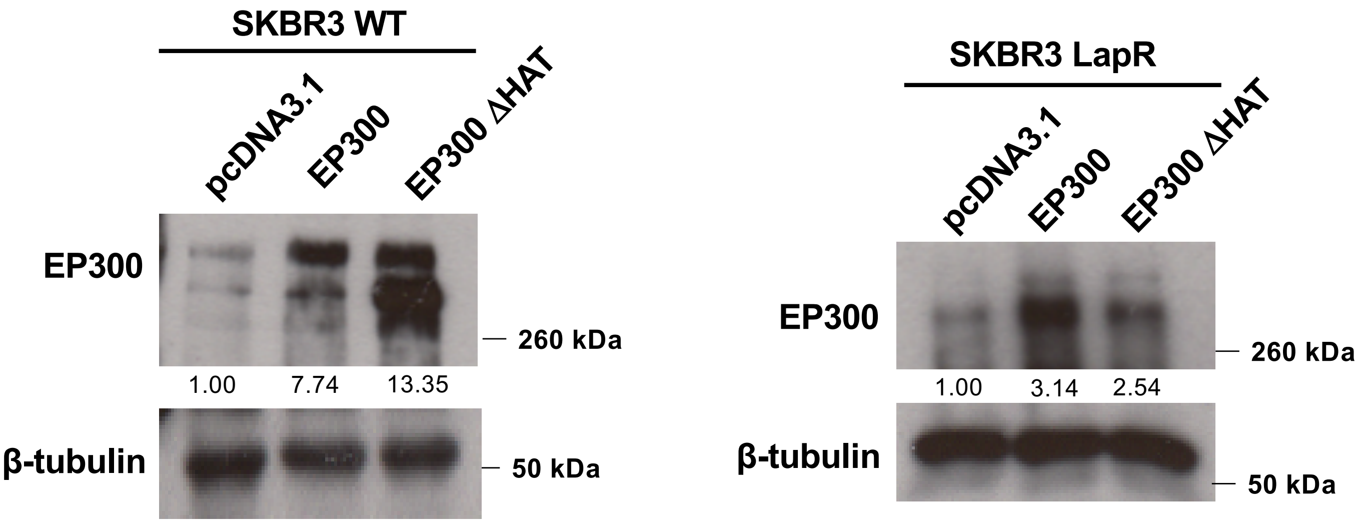

Supplementary Figure S8J
Protein quantification is performed by ImageJ analysis from Figure S7B

Supplementary Fig. S8K

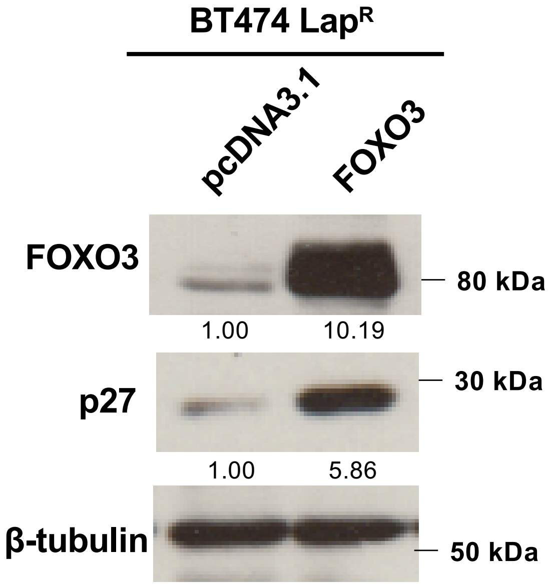

Supplementary Figure S8K
Protein quantification is performed by ImageJ analysis from Figure S2A

Supplementary Fig. S8L

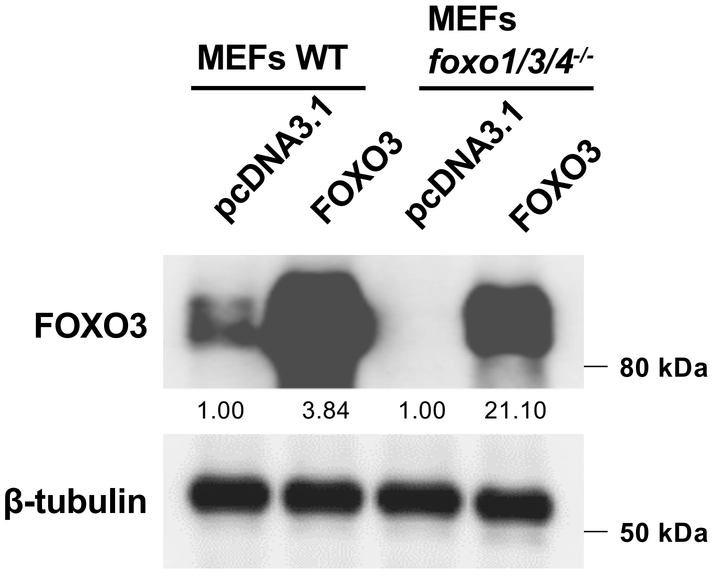

Supplementary Figure S8L
Protein quantification is performed by ImageJ analysis from Figure S2B

Supplementary Fig. S8M

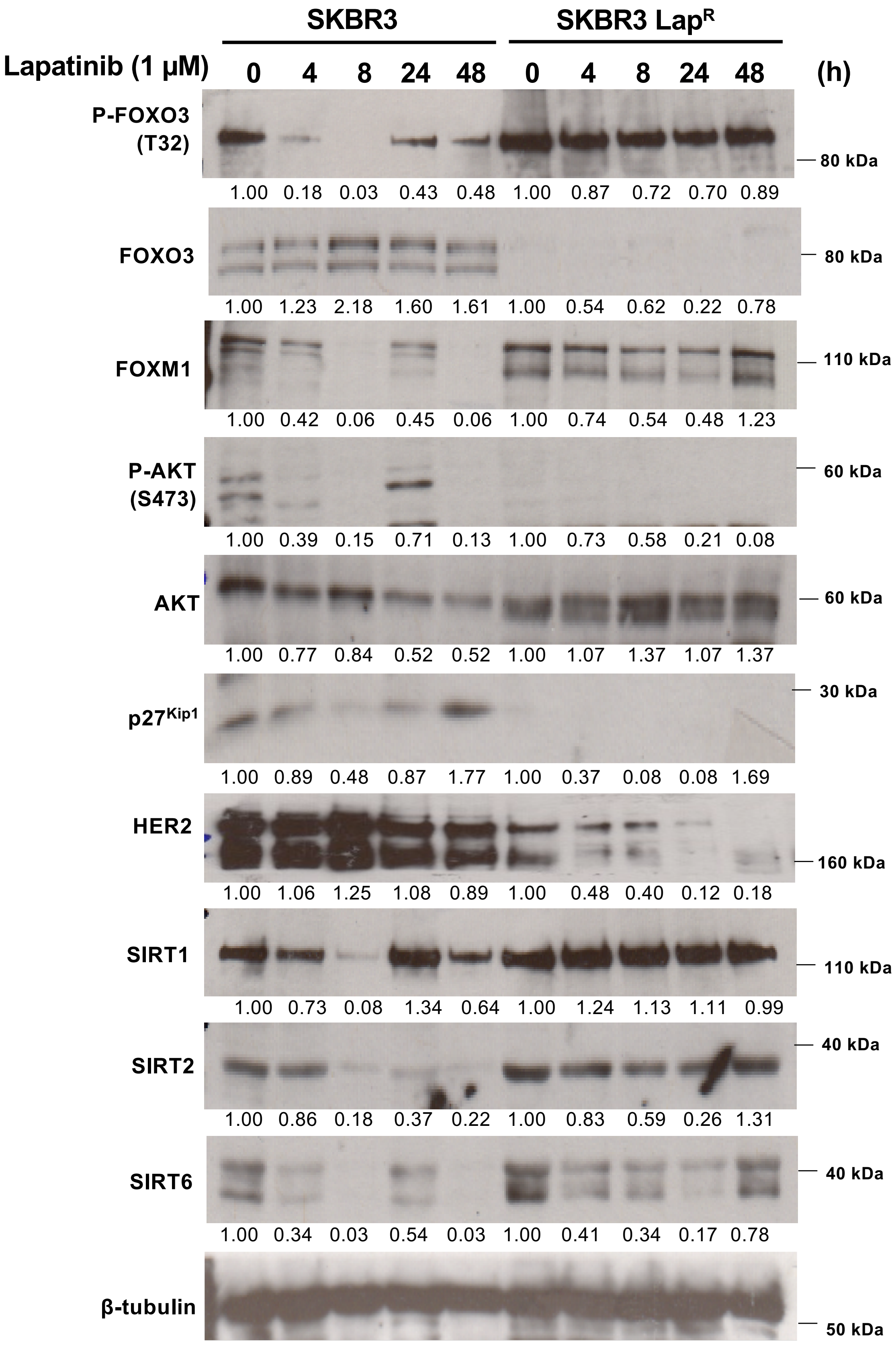

Supplementary Figure S8M

Protein quantification is performed by ImageJ analysis from Figure S4A
